# Synchronized Retrovirus Fusion in Cells Expressing Alternative Receptor Isoforms Releases the Viral Core into Distinct Sub-cellular Compartments

**DOI:** 10.1371/journal.ppat.1002694

**Published:** 2012-05-10

**Authors:** Sergi Padilla-Parra, Mariana Marin, Naoyuki Kondo, Gregory B. Melikyan

**Affiliations:** 1 Division of Pediatric Infectious Diseases, Emory University Children's Center, Atlanta, Georgia, United States of America; 2 Children's Healthcare of Atlanta, Atlanta, Georgia, United States of America; Fred Hutchinson Cancer Research Center, United States of America

## Abstract

Disparate enveloped viruses initiate infection by fusing with endosomes. However, the highly diverse and dynamic nature of endosomes impairs mechanistic studies of fusion and identification of sub-cellular sites supporting the nucleocapsid release. We took advantage of the extreme stability of avian retrovirus-receptor complexes at neutral pH and of acid-dependence of virus-endosome fusion to isolate the latter step from preceding asynchronous internalization/trafficking steps. Viruses were trapped within endosomes in the presence of NH_4_Cl. Removal of NH_4_Cl resulted in a quick and uniform acidification of all subcellular compartments, thereby initiating synchronous viral fusion. Single virus imaging demonstrated that fusion was initiated within seconds after acidification and often culminated in the release of the viral core from an endosome. Comparative studies of cells expressing either the transmembrane or GPI-anchored receptor isoform revealed that the transmembrane receptor delivered the virus to more fusion-permissive compartments. Thus the identity of endosomal compartments, in addition to their acidity, appears to modulate viral fusion. A more striking manifestation of the virus delivery to distinct compartments in the presence of NH_4_Cl was the viral core release into the cytosol of cells expressing the transmembrane receptor and into endosomes of cells expressing the GPI-anchored isoform. In the latter cells, the newly released cores exhibited restricted mobility and were exposed to a more acidic environment than the cytoplasm. These cores appear to enter into the cytosol after an additional slow temperature-dependent step. We conclude that the NH_4_Cl block traps the virus within intralumenal vesicles of late endosomes in cells expressing the GPI-anchored receptor. Viruses surrounded by more than one endosomal membrane release their core into the cytoplasm in two steps – fusion with an intralumenal vesicle followed by a yet unknown temperature-dependent step that liberates the core from late endosomes.

## Author Summary

Endosomal trafficking and regulation of retrovirus fusion is poorly understood, due in part to heterogeneity of viral particles and their asynchronous entry into an endocytic pathway. Here, we used an avian retrovirus that enters host cells in a receptor- and low pH-dependent manner. This feature allowed capturing the virus in intracellular compartments through raising the endosomal pH. Virus fusion was synchronously initiated upon permitting endosome acidification and visualized in real-time by single particle imaging. We found that different receptor isoforms directed virus into distinct sub-cellular compartments supporting quick release of the viral core. Through tracking individual sub-viral particles released from endosomes, we found that the full length receptor mediated core delivery into the cytoplasm. By contrast, fusion mediated by the GPI-anchored receptor released the core into another endosomal compartment, from which the core entered the cytosol through an additional temperature-dependent step. These findings demonstrate different permissiveness of endosomal compartments to viral fusion and the existence of a post-fusion step leading to the cytosolic release of cores and initiation of infection.

## Introduction

A large number of enveloped and non-enveloped viruses enter cells through endocytosis [1]. Depending on the nature of cellular receptors and replication strategies, viruses have evolved to utilize alternative entry routes and fuse with distinct intracellular compartments. Preferential entry from early or late endosomes is achieved through adjusting the pH threshold for triggering fusion [2]–[4] or by relying on endosome-specific factors, such as lipids [5]–[7] or lysosomal enzymes [8], [9]. There is evidence for complex regulation of early and late steps of viral fusion. For instance, viruses, which are activated by mildly acidic pH in early endosomes, may require late endosome-resident factors to complete their fusion process and release the nucleocapsid [5], [7]. In other words, virus-endosome fusion and capsid release into the cytosol could be spatially and temporally separated. The asynchronous and often rate-limiting steps of virus internalization and trafficking hamper the studies of endosomal fusion and its regulation. In order to gain mechanistic insights into the virus-endosome fusion, it is essential to isolate the virus fusion step from the upstream asynchronous processes and to control the timing of low pH exposure and acidity of endosomal compartments.

Avian Sarcoma and Leukosis virus (ASLV) initiates fusion *via* a two-step mechanism that involves priming of Env glycoprotein by cognate receptors (presumably on the cell surface) and low pH-dependent fusion with endosomes [3], [10]–[12]. The receptor priming of Env confers the competence for acid-mediated refolding that drives the merger of viral and endosomal membranes. Subtype A ASLV infects cells expressing either of the alternative isoforms of the TVA receptor, TVA800 and TVA950, which reside in lipid rafts and liquid-disordered domains, respectively [13]–[15]. Importantly, the transmembrane (TVA950) and GPI-anchored (TVA800) isoforms appear to direct the virus entry through distinct endocytic pathways [16]. ASLV fuses more efficiently with TVA950-expressing than with TVA800-expressing cells [17]. Considering that these isoforms have identical ectodomains [13], [14] and exhibit a similar glycosylation pattern (data not shown), the difference in fusion efficiency is likely due to the properties of sub-cellular compartments harboring the virus at the time of fusion. A critical feature of ASLV is the extreme stability of the Env-receptor complexes at neutral pH [11], [16], [18], which enables the virus to survive the prolonged fusion block imposed upon raising the endosomal pH by NH_4_Cl. Removal of NH_4_Cl after several hours of incubation restores acidic conditions and quickly initiates infection [16]. This arrest/release protocol thus allows one to isolate virus-endosome fusion from the preceding internalization and trafficking steps [16], [17].

Here, we employed the NH_4_Cl arrest/release protocol to synchronize the ASLV fusion. Removal of weak base resulted in an immediate and uniform pH drop in all intracellular compartments, thus standardizing the fusion trigger. Synchronized fusion facilitated the detection of viral core release from an endosome as a result of full enlargement of the fusion pore. We defined the sites of core release in cells expressing alternative receptors by (i) measuring the mobility of sub-viral particles after spatial separation from endosomes and (ii) probing the dynamics of pH changes within the recipient compartments upon removal of NH_4_Cl. These experiments showed that the viral cores were released into the cytosol of TVA950 cells. By contrast, the cores were delivered into endosomal compartments of TVA800 cells, from which they entered into the cytoplasm after an additional slow temperature-dependent step. Our findings imply that ASLV fusion is redirected to distinct intracellular compartments of cells expressing alternative receptors in the presence of NH_4_Cl. This approach provides new insights into the morphology and fusion-permissiveness of these compartments.

## Results

### Rationale

Removal of ammonium chloride results in acidification of all intracellular compartments, including the cytoplasm [19]. This effect is due to protons left behind by NH_4_^+^, which traverses a membrane in its neutral NH_3_ form (Figure S1A). Here, we synchronized ASLV fusion by allowing virus internalization in the presence of NH_4_Cl followed by removal of the fusion block. A quick drop of endosomal pH caused by the NH_4_Cl removal thus serves as a standardized trigger for ASLV-endosome fusion, irrespective of the distinct trafficking itineraries for this virus through TVA800 and TVA950 isoforms [16], [17]. This “on demand” ASLV fusion with endosomal compartments offers several advantages for single particle imaging, since it (i) bypasses the slow pre-fusion steps and likely allows optimal priming of Env by receptors; (ii) eliminates the differences in the pH within distinct intracellular compartments; and (iii) simplifies virus tracking. In addition, the NH_4_Cl arrest redirects the virus entry to distinct intracellular compartments thus providing an opportunity to probe their relative permissiveness to viral fusion. As we will show below, although NH_4_Cl impairs endosomal trafficking and maturation [20]–[22], these conditions provide valuable information regarding viral fusion with alternative sub-cellular sites and elucidate early post-fusion steps.

### Removal of NH_4_Cl causes quick and uniform acidification of all intracellular compartments

We first assessed the effect of NH_4_Cl removal on endosomal pH by loading intracellular compartments with the endosomal pH indicator, pHrodo dextran [23]. In the presence of weak base, the endosomal pH exhibited a fairly narrow distribution around a neutral value (Figure S1B). Immediately after NH_4_Cl removal by local perfusion with Hank's buffer (HBSS), the endosomes became uniformly acidic (Figure S1B, C).

We next measured the pH changes within virus-carrying endosomes by co-labeling the viral envelope with the GFP-ICAM-1 chimera [17] and the membrane dye, DiD. Viral particles were identified based on the interior marker, MLV Gag-mKO [24]. Triple-labeled pseudoviruses were internalized by cells upon incubation in the presence of NH_4_Cl. Since the majority of ASLV is internalized by CV-1-derived target cells within 40 min [17], we choose this time interval for NH_4_Cl pretreatment. Acidification of endosomal lumen upon removal of NH_4_Cl quenched the GFP fluorescence (pKa 6.15 [25]), while the pH-independent DiD probe served as a reference signal for the ratiometric pH measurements (Figure S1D-F). We found that endosomal pH exponentially decayed throughout the 2 min-perfusion with HBSS reaching 5.7±0.3 (n = 20) in both TVA800 and TVA950 cells at the end of the pulse (Figure S2A).

In parallel experiments, the changes in the cytosolic pH were monitored by labeling the membrane of GFP-transfected CV-1 cells with DiD and measuring the ratio of the intracellular GFP and DiD fluorescence upon addition/removal of NH_4_Cl. As shown in Figure S2B, the cytosolic pH dropped to ∼6.8 at the beginning of HBSS perfusion, but nearly completely recovered before the cells were returned to NH_4_Cl. This likely occurs as a result of compensatory mechanisms that effectively raise the intracellular pH [19], [26], [27]. To conclude, the NH_4_Cl arrest/release protocol results in quick and uniform acidification of endosomes and the cytosol. This feature is essential for inducing fusion of internalized ASLV irrespective of the identity of virus-bearing endosomes in TVA800 and TVA950 cells.

### ASLV fuses synchronously with endosomes upon lifting the NH_4_Cl block

We have previously shown that labeling of pseudoviruses with MLV Gag-GFP permits visualization of single virus fusion in live cells [17]. Gag-GFP is cleaved into defined fragments upon virus maturation, including the GFP-tagged nucleocapsid protein. This fragment is loosely trapped within mature viruses and is readily released upon permeabilization of the viral membrane or as a result of fusion [28]. Our single virus fusion assay is thus based on the loss of GFP content from a viral particle. By contrast, the membrane marker, DiD, undergoes a limited dilution due to its redistribution into an endosomal membrane and thus retains its punctate appearance [17], [29].

**Figure 1:**
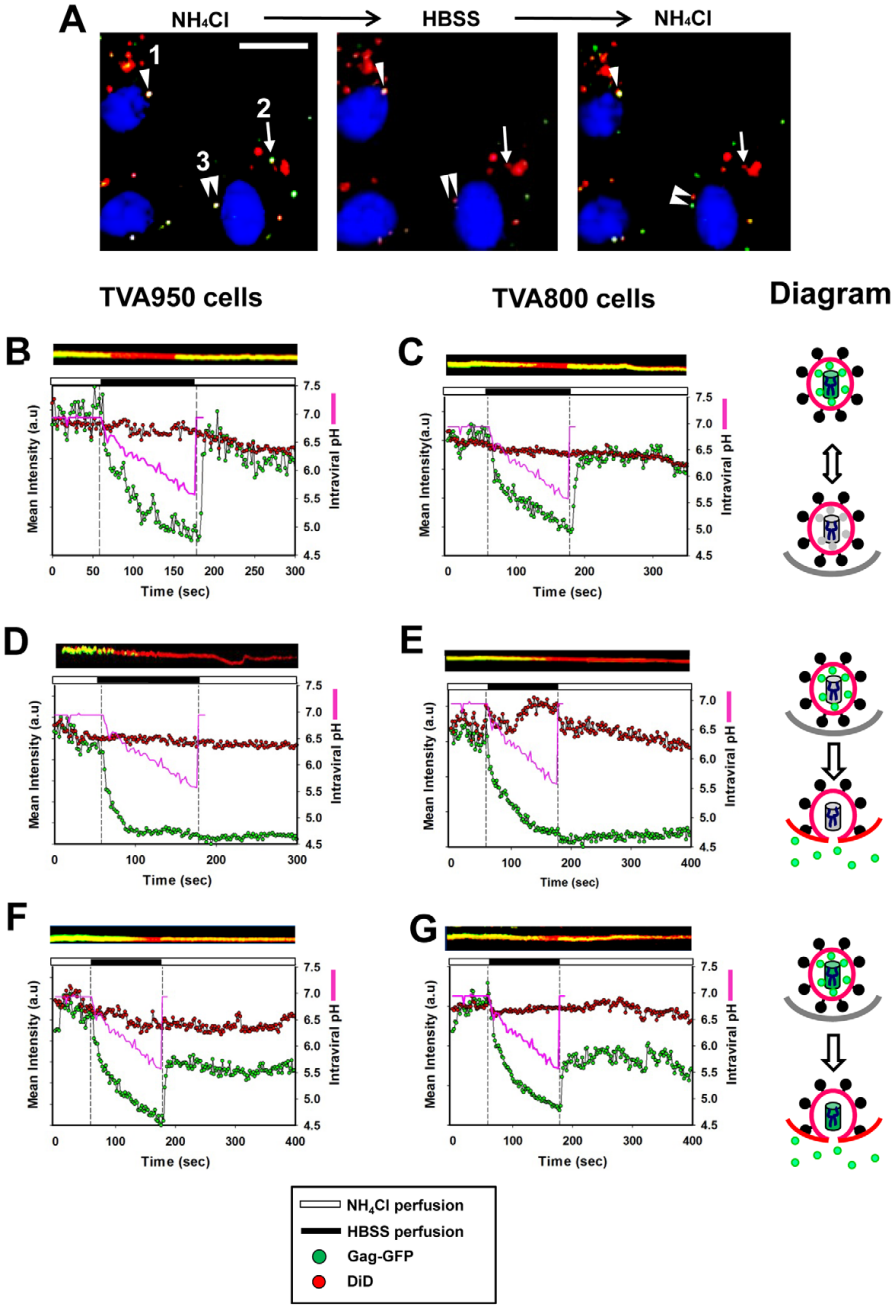
Arrest and synchronous triggering of ASLV pseudovirus fusion with endosomes using NH_4_Cl. (A) Micrograph showing single ASLV pseudoviruses co-labeled with Gag-GFP (green) and DiD (red) following the incubation with cells expressing TVA800 for 40 min at 37°C in isotonic HBSS supplemented with 70 mM NH_4_Cl (left panel). Removal of NH_4_Cl through perfusion with HBSS caused a marked decrease in the GFP signal, but not in DiD fluorescence (middle). Upon returning to NH_4_Cl (right), the GFP fluorescence of fusion-incompetent particles fully recovered (#1, arrowhead), whereas the signal from fused particles remained undetectable (#2, arrow). The viral core release into the cytosol is manifested in spatial separation of green and red puncta (#3, double arrowhead). Cell nuclei are labeled with Hoescht (blue). Scale bar is 15 µm. (B–G) ASLV pseudoviruses co-labeled with Gag-GFP and DiD were internalized by CV-1 cells expressing TVA950 (B, D, F) or TVA800 (C, E, G) in the presence of NH_4_Cl, and virus-endosome fusion was initiated by perfusion with HBSS, as described in Materials and Methods. (B, C) The fluorescence intensity profiles corresponding to particles that failed to fuse following the arrest/release protocol, as evidenced by complete recovery of the GFP signal. (D, E) Examples of complete loss of the GFP marker from virions. (F, G) Partial release of the content marker. Cells were initially perfused with 70 mM NH_4_Cl in HBSS (white thick horizontal bars at the top of each graph) followed by perfusion with plain HBSS for 2 min (black horizontal bars) and returned to NH_4_Cl. The mean intensities of GFP and DiD fluorescence from single particles are shown (green and red circles, respectively). Pink lines show the changes in the intraviral pH upon removal of NH_4_Cl (for details, see Figures. S1 and S2). Kymographs illustrating the time-dependent changes in the mean GFP and DID signals (overlaid) are also shown above each plot. An irreversible transition from yellow (co-localized GFP and DiD signals) to red (DiD only) manifests the release of the GFP-based content marker into the cytosol. Diagrams of the fusion outcomes (reversible GFP quenching by acidic pH, full and partial release of the content marker) for respective panels are shown on the right.

To synchronize the ASLV fusion, Gag-GFP and DiD labeled pseudoviruses were pre-bound to target cells in the cold and incubated at 37°C in the presence of NH_4_Cl. Cells were mounted on a heated microscope stage and imaged in an NH_4_Cl-containing buffer for a short period of time before removing the fusion block by perfusion with HBSS. Internalized ASLV particles that failed to undergo fusion with endosomes following the NH_4_Cl arrest/release protocol exhibited reversible GFP quenching-dequenching pattern (Figure 1A–C, and video S1) reflecting changes in the intraviral pH (Figure S2C). Three types of fusion-related events were induced by the arrest/release protocol in either TVA950 or TVA800 cells (Table 1). First, nearly 20% of virions fully released their content as a result of fusion (Figure 1D, E and video S2). Second, about the same fraction of viruses partially lost their content marker (Figure 1F, G). A partial loss of viral content could be due to incomplete cleavage of the Gag-GFP precursor, which would remain in large oligomeric complexes, unable to permeate through a small fusion pore. Consistent with this notion, a similar fraction of viruses (around 20%) exhibited a partial loss of the GFP marker following the saponin-mediated lysis (Figure S3). Third, we observed a combination of a partial GFP release with subsequent separation of the GFP-tagged core from the viral/endosomal membrane labeled with DiD (Table 1 and Figure 1A, double arrowhead).

**Table 1:** Outcomes of synchronized ASLV-endosome fusion.

	Full release	Partial Release	Core release	No fusion
TVA800 cells (n = 91)	18.7%	26.3%	12.1%	42.9%
TVA950 cells (n = 90)	18.6%	19.8%	13.2%	48.4%

The relative frequency of fusion outcomes was determined by pooling data from at least 4 independent experiments.

Irrespective of the TVA isoform, all three fusion-related events collectively accounted for ∼50% of viruses internalized in the presence of NH_4_Cl (Table 1). By comparison, 16% and 4% of viruses fused with TVA950 and TVA800 cells, respectively, through conventional entry in the absence of NH_4_Cl [17]. Thus, the arrest/release protocol markedly enhanced the efficiency of ASLV fusion, especially with TVA800 cells. The enhancing effect was likely due to several factors, including the quick acidification of endosomal lumen, and the ease of detection of synchronous fusion events within arrested endosomes. More importantly, the markedly improved probability of fusion could be due to a higher fusion-permissiveness of endosomes harboring the virus in the presence of NH_4_Cl compared to sites of uninterrupted ASLV fusion.

### Synchronous initiation of ASLV fusion allows detection of early post-fusion events

**Figure 2:**
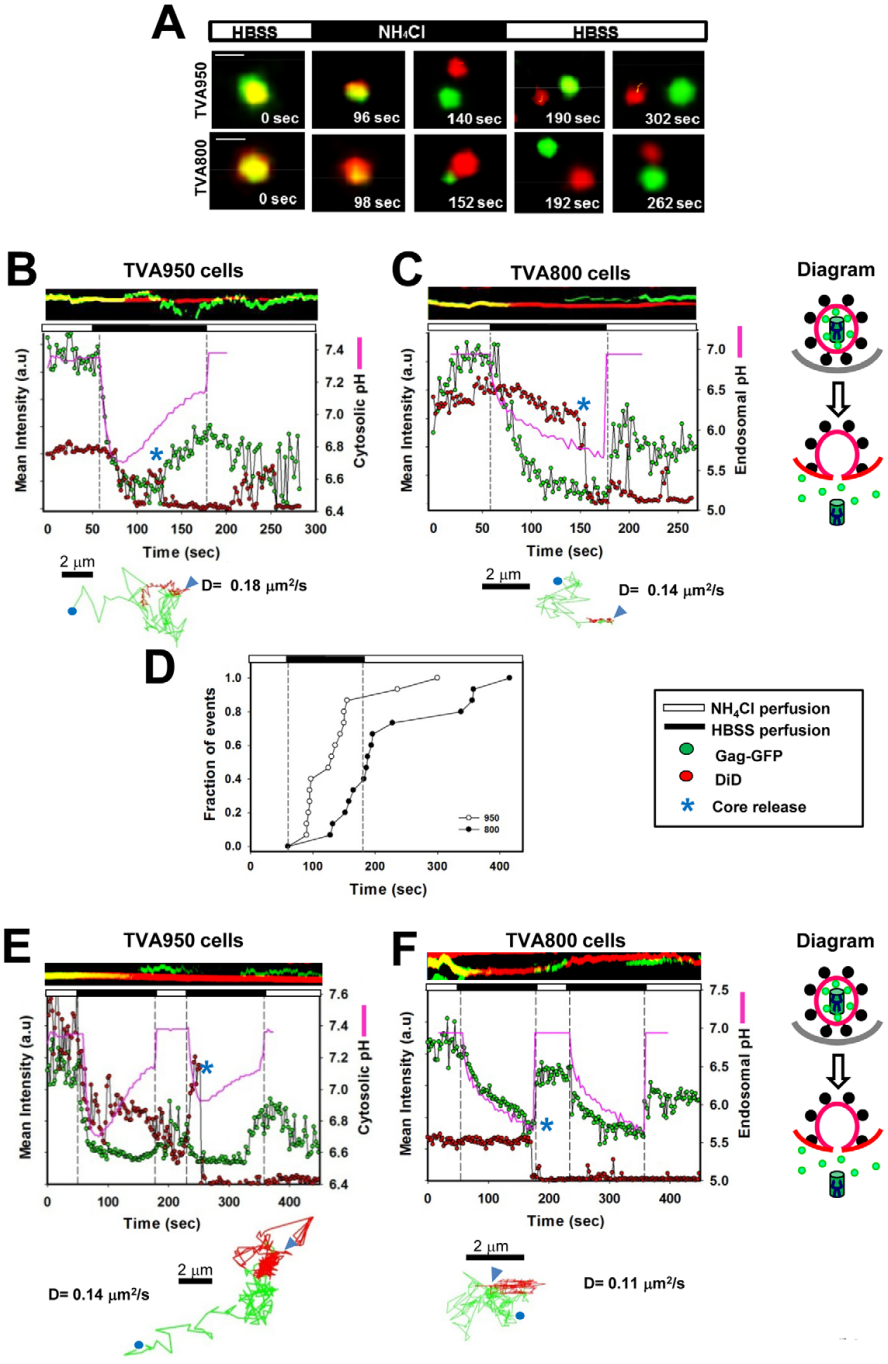
Spatial separation of the GFP-tagged viral cores from membranes as a result of fusion. (A) Spatial separation of the GFP- and DiD-tagged puncta following the NH_4_Cl arrest/release protocol. Double-labeled viruses were internalized by TVA950 (top panel) and TVA800 (bottom panel) cells in the presence of NH_4_Cl. Fusion was triggered by replacing NH_4_Cl with HBSS for 2 min (black horizontal bars) through local perfusion, and cells were returned to NH_4_Cl. Following the initial decay in the GFP fluorescence due to a partial release of the content marker, co-labeled particles split in two and continued to drift apart. The GFP signal partially recovered during the HBSS perfusion in TVA950 cells, but not TVA800 cells where recovery occurred only after returning to NH_4_Cl. Scale bar is 0.15 µm. (B, C) Separation of green (Gag-GFP) and red (DiD) puncta in NH_4_Cl-arrested TVA950 (B, video S3) and TVA800 (C, video S4) cells during perfusion with HBSS. The graphs show changes of the mean intensity of green and red signals for single particles shown in panel A. A drop in the red signal during the HBSS perfusion (marked by a blue asterisk) occurs due to the separation of formerly co-localized red and green puncta. The changes in cytosolic (B, E) and endosomal (C, F) pH during the HBSS perfusion measured in separate experiments (for details, see Figure S2) are shown by pink lines. (D) Kinetics of spatial separation of SVPs and the viral membrane triggered by a 2 min-perfusion with HBSS (black horizontal bar and vertical dashed lines). Cumulative probabilities of core release as a function of time are plotted for TVA800 (filled circles) and TVA950 (open circles) cells. (E, F) Late spatial separation events occurring at the end or after the first HBSS pulse (horizontal black bars above each plot). The SVP release in a TVA950 cell (E, asterisk) was followed by partial recovery of the Gag-GFP fluorescence during the second HBSS perfusion, which paralleled the increase in the cytosolic pH (pink line). A spike in the DiD signal at the beginning of the second HBSS pulse in panel D occurred due to a transient overlap between separated puncta. The late SVP release events in TVA800 cells (F) did not exhibit the Gag-GFP signal recovery in the course of HBSS perfusion. The two-dimensional trajectories of the recipient endosomes (red) and the GFP-tagged sub-viral particles (green) obtained by single particle tracking are shown under for each respective panel. The beginning and the end of trajectories are marked by triangles and circles, respectively. A diagram showing the viral content and core release (spatial separation from the membrane) as a result of fusion are shown on the right.

As shown above, ∼20% of pseudoviruses released only a fraction of their GFP marker as a result of fusion (Figure 1F, G). This was likely due to a partial cleavage of Gag-GFP during virus maturation. The presence of particles that contained both a releasable GFP maker and uncleaved Gag-GFP provides an opportunity to monitor the formation and enlargement of Env-mediated fusion pores, respectively. Indeed, a sizeable fraction of events were manifested in a diminution of the GFP signal upon perfusion with HBSS/NH_4_Cl followed by spatial separation of green and red puncta (Figures 1A and 2A). These events occurred with similar frequencies in TVA950 and TVA800 cells: 13.9±5% (n = 87) and 12.3±5% (n = 86), respectively (Table 1). Upon separation from endosomes, which retained the viral DiD marker, GFP-tagged puncta exhibited accelerated motion and traveled as one entity without losing fluorescence intensity (Figure 2, trajectories shown below the graphs, and videos S3 and S4). These results further support the notion that green puncta are sub-viral particles (SVPs) containing unprocessed Gag-GFP. We analyzed the spatial separation events by tracking the GFP-labeled particles. The moment of spatial separation was defined based on the loss of the DiD signal which was initially co-localized with the GFP signal (Figure 2, blue asterisks).

In contrast to the synchronized ASLV fusion, we very rarely observed spatial separation of core and membrane markers after a partial loss of a diffusible content marker upon the virus entry through a conventional route. This is likely due to the delayed release of the viral core, which could not be reliably detected because of the loss of the DiD signal (data not shown). The disappearance of DiD marker after redistribution to an endosome probably occurs through its trafficking out of an endosome [17], [29]. The lack of detectable capsid release through uninterrupted ASLV entry suggests that Env-mediated fusion pores enlarge slowly, so the capsids are released at later times after trafficking to different compartments. It appears that, in the presence of NH_4_Cl, ASLV is redirected to endosomes that support quick pore enlargement and core release.

### The rate of fusion pore dilation depends on the receptor isoform

Regardless of whether or not the released SVPs corresponded to the *bona fide* viral capsid or to large oligomeric Gag-GFP complexes, their liberation signifies pore dilation and can thus be used to study this final step of fusion. Similar probabilities of the pore formation (loss of diffusible GFP) and release of SVPs in cells expressing TVA800 and TVA950 (∼13%, Table 1) indicate that, under our conditions, both receptor isoforms support equally efficient pore enlargement. We then asked whether the rate of pore enlargement was also independent of the receptor isoform. The time course of pore dilation was deduced from the lag between the onset of HBSS perfusion, which quickly triggered the pore opening, and spatial separation of SVPs from endosomes, as determined by the loss of the DiD signal from double-labeled particles (Figure 2B and C (blue asterisks), and videos S3 and S4, respectively). The obtained lag times between the onset of HBSS perfusion and SVP release were plotted as cumulative probabilities over time (Figure 2D). This analysis revealed that the SVPs were released (and thus the pore enlarged) faster in TVA950 cells compared to cells expressing the GPI-anchored receptor. Approximately 83% of these events were detected within the 2 min window of HBSS perfusion for TVA950 cells, whereas only 40% of cores were released into TVA800 cells within this interval.

Together, our results imply that, whereas the arrest/release protocol promotes efficient opening and expansion of fusion pores in cells expressing either receptor isoform, the apparent rate of pore dilation is faster in TVA950 cells (Figure 2D). The more efficient dilation of pores formed in TVA950 cells compared to TVA800 cells is consistent with our previous observation that early fusion pores formed by ASLV are larger in cells expressing TVA950 compared to TVA800 under normal entry conditions [17]. While dissociation of the viral core from the membrane could itself be a slow process [30], the receptor isoform-dependence of the release kinetics argues against this possibility. On the other hand, we sometimes detected incomplete separation of GFP-tagged sub-viral particles from the viral membrane which could be due to partial pore dilation (Figure S4).

### TVA isoforms mediate release of viral cores into distinct subcellular compartments

Next we sought to define the cellular compartments into which the SVPs were released. We reasoned that the formation/enlargement of a fusion pore between the virus and the limiting membrane of an endosome should bring the intraviral pH to that of the cytosol. Since the cytosolic and endosomal/intraviral pH exhibit markedly different profiles following the removal of NH_4_Cl (Figure S2), the formation of a fusion pore with the limiting membrane of an endosome is expected to raise the intraviral pH. By contrast, if released cores remain inside an endosome (see Discussion for the model), the core-associated GFP will be exposed to a continuously dropping pH throughout the HBSS pulse (Figure 1B, C, pink lines).

We found that SVPs released into TVA950 cells during the HBSS perfusion typically exhibited pH responses that were consistent with their release into the cytosol. The GFP signal initially dropped due to the acid-mediated quenching and the release of a diffusible fraction of this marker. However, green fluorescence partially recovered before the end of HBSS perfusion (Figure 2B, pink lines), in spite of the continued decrease of endosomal pH (Figures 2C, pink line, and S2A). The partial recovery of the GFP fluorescence occurred at the time or soon after spatial separation from an endosome and paralleled the increase in cytosolic pH (Figure 2B, video S3). These results imply that SVPs are released into the cytosol of TVA950 cells. In sharp contrast, spatial separation events in TVA800 cells were not accompanied by recovery of the GFP signal during the HBSS pulse (Figure 2C, videoS4), consistent with the continuous drop of endosomal pH (pink line). Fluorescence partially recovered only after returning to NH_4_Cl. This pattern was observed for nearly all spatial separation events in TVA800 cells (n = 18, see also Figures S5 and S6), suggesting that cores entered intracellular compartments distinct from the cytoplasm. It is worth pointing out that SVPs were not always released into the cytosol of TVA950 cells: in 3 out of 14 spatial separation events the GFP fluorescence decayed monotonously throughout the HBSS perfusion and partially recovered only after returning to NH_4_Cl (exemplified in Figure S7). In other words, the ASLV fusion in NH_4_Cl-arrested TVA950 cells did not uniformly result in the viral content release into the cytosol.

As shown in Figure 2D, a fraction of SPVs were released after the end of the HBSS pulse. Since these delayed events took place under conditions when pH in all intracellular compartments was raised to neutrality by NH_4_Cl, the sites of core release could not be unambiguously established. To address this issue, a second HBSS pulse was applied. During the second pulse, the signal from SVPs in TVA950 cells exhibited a biphasic behavior similar to that of the cytosolic pH (Figure 2E, n = 11), which was consistent with the delivery of viral cores into cytoplasm. By comparison, the GFP signal from the late SVPs release events in TVA800 cells remained quenched throughout HBSS perfusion and increased only after returning to NH_4_Cl (Figure 2F, n = 6). Collectively, our results indicate that, in cells expressing the GPI-anchored TVA isoform, ASLV Env mediates the release of SPVs into compartments that lack the active buffering capacity of the cytoplasm.

**Figure 3:**
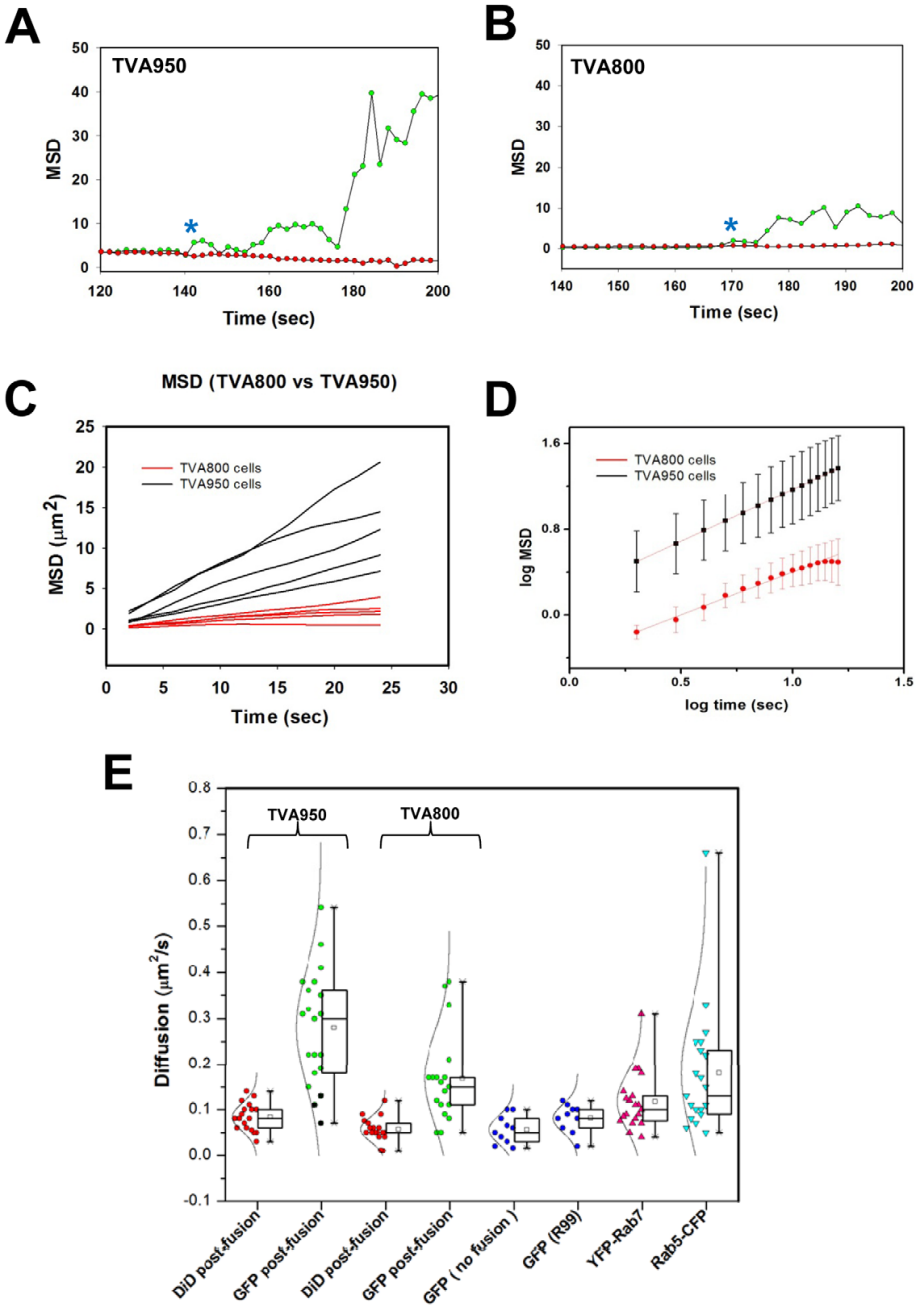
Sub-viral particles released by ASLV-endosome fusion exhibit increased mobility. (A, B) Examples of the mean square displacement (MSD) as a function of time for GFP-tagged (green circles) and DiD-tagged (red circles) particles in TVA950 (A) and TVA800 (B) cells. Blue asterisks mark spatial separation of cores from membranes that coincided with an abrupt increase in the MSD slope for SVPs (green), while the movement pattern of DiD-recipient endosomes did not change significantly. (C) Representative MSD curves after SVP release are shown for five SVPs in TVA950 cells (black lines) and in TVA800 cells (red lines). (D) Log-log plot showing the mean MSD slopes and standard deviations for 15 SVP trajectories after core release in TVA950 (black) and TVA800 (red) cells each. (E) Distributions of diffusion coefficients for endosomes and viral particles before and after SVP release. Diffusion coefficients were calculated, as detailed in the Materials and Methods. Box plots together with the Gaussian distribution for the diffusion values (circles and solid lines) corresponding to 18 endosomes (“DiD post-fusion”, red circles) and SVPs (“GFP post-fusion”, green circles) after core release are shown for TVA800 and TVA950 cells. Diffusion coefficients of three SVPs that were judged to be released into endosomal compartments of TVA950 cells are colored black (under “GFP post-fusion”). For comparison, the motility of 10 non-fusogenic particles trapped in endosomes after perfusion with HBSS is shown (“GFP, no fusion”, yellow circles). In addition, the diffusion coefficients for 10 not fused particles in the presence of the inhibitory peptide R99 is shown (blue circles). The diffusion coefficients of endosomes tagged with YFP-Rab7 (n = 20) and CFP-Rab5 (n = 20) in TVA800 and TVA950 cells (pooled data) are shown by magenta and cyan triangles, respectively.

Viral cores that entered an endosome are expected to be restricted in their motion. We therefore compared the motion patterns of the newly release SVPs in cells expressing alternative receptor isoforms. We found that motion of virus-carrying endosomes in NH_4_Cl-arrested cells was restricted, as evidenced by a shallow slope of the mean square displacement (MSD) over time (Figure 3A, B, red circles). However, coincident with the time of spatial separation (blue asterisks), the initial MSD slope for green puncta drastically increased (Figure 3A, B, green circles), whereas the movement of DiD-positive puncta (host endosome) continued to be restricted. For the liberated SVPs, the average slopes of log(MSD) vs. log(time) were 0.98±0.003 and 0.80±0.01 (n = 15) in TVA950 and TVA800 cells, respectively (Figure 3C, D). The MSD slopes close to 1 are indicative of free diffusion of SVPs released as a result of fusion [31].

To further characterize the motion patterns of these particles, we calculated the effective diffusion coefficients (D) from the MSD plots for endosomes, viruses trapped in endosomes, and for liberated sub-viral particles. This analysis confirmed that endosomes and viruses residing within endosomes tended to move slowly (typical D below 0.1 µm^2^/sec, Figure 3E, in accordance to [32]). Only a fraction of Rab5-positive endosomes exhibited significant mobility. In contrast to endosomes, released SVPs were relatively more mobile. Interestingly, SVPs in TVA950 cells moved nearly twice as fast as in TVA800 cells: D = 0.30±0.1 (n = 20) and 0.16±0.09 µm^2^/sec (n = 20), respectively (P<0.0005). The fact that movement of SVPs released into TVA800 cells was more restricted than in TVA950 cells is consistent with delivery of these particles into endosomal compartments. Analysis of individual particle trajectories confirmed this finding (Figures S5 and S6). On the other hand, a few SVPs that did not exhibit changes in the GFP signal paralleling the pH changes in the cytosol of TVA950 cells (e.g., Figure S7) also moved slowly, consistent with their release into endosomes (Figure 3E, black circles). To conclude, single particle tracking further supported our conclusion that viral cores were released into distinct compartments within the NH_4_Cl-arrested TVA800 and TVA950 cells.

### Viruses internalized through TVA isoforms in the presence of NH_4_Cl are trapped within different endosomes

How could the apparent lack of SVP release into the cytoplasm of TVA800 cells be rationalized? Putative compartments harboring SVPs must be relatively large, as suggested by the disappearance of the GFP signal occurring as a result of significant dilution of the content marker. In agreement with this notion, in TVA800 cells the viral cores traveled considerable distances from endosomes with which they fused. Within a few minutes after SPV release the maximum distance between liberated SVPs and the endosome of origin reached 4.9±1.7 µm (n = 10) and 11.3±4.2 µm (n = 10) in TVA800 and TVA950 cells, respectively. The markedly different (P<0.001) separation of SVPs and endosomes in these cells is another manifestation of the confined core movement in TVA800 cells. These findings are thus in line with the notion that SVPs are released into micron-size subcellular compartments of NH_4_Cl-arrested TVA800 cells.

**Figure 4:**
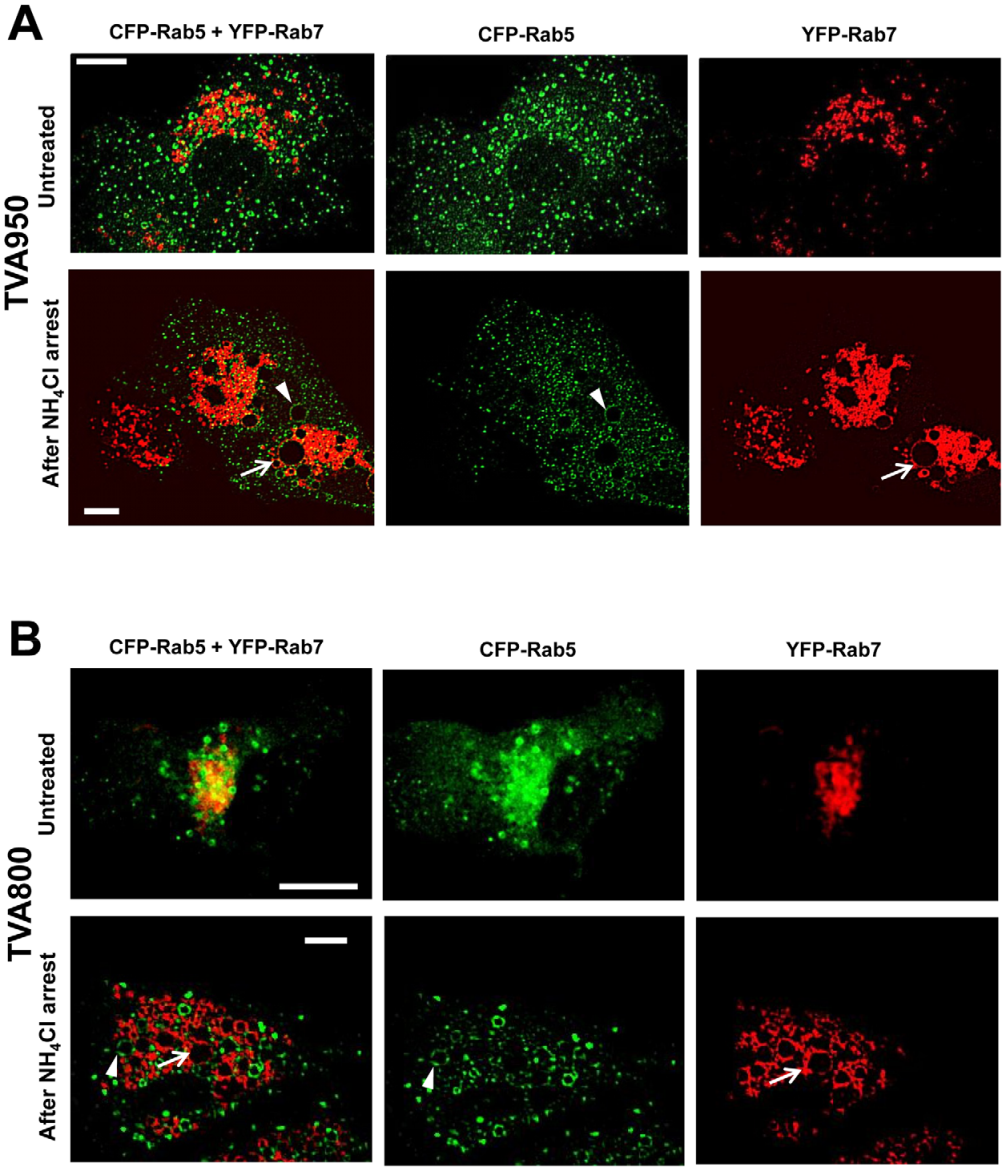
NH_4_Cl arrest creates large endosomes. (A) TVA950 cells co-expressing CFP-Rab5 (green) and YFP-Rab7 (red) before (top row) and after (bottom row) incubation with 70 mM NH_4_Cl for 40 minutes at 37°C. Enlarged endosomes positive for Rab5 (diameter 12 µm) and Rab7 (diameter 15 µm) are indicated by arrowheads and arrows, respectively. Scale bars are 20 µm. (B) Same as in panel A, but for TVA800 cells. Enlarged endosomes positive for Rab5 (diameter 3.5 µm) and Rab7 (diameter 5 µm) are indicated by arrowheads and arrows, respectively. Scale bar 15 µm.

In order to test whether such unusually large compartments exist in CV-1 cells expressing either receptor, we transiently expressed markers for early (CFP-Rab5) and late (YFP-Rab7) endosomes in TVA800 and TVA950 cells. Figure 4 shows that, whereas these markers were not usually associated with large endosomes in untreated cells, large circular compartments reaching 3–4 µm in diameter became apparent in both cell lines following pre-incubation with NH_4_Cl. Live cell imaging revealed that large vacuoles started to form as early as after 10 min at 37°C in the presence of NH_4_Cl (data not shown). It is therefore possible that small ASLV-carrying endosomes are somehow delivered into these abnormally large compartments in NH_4_Cl-arrested TVA800, but not in TVA950 cells.

**Figure 5:**
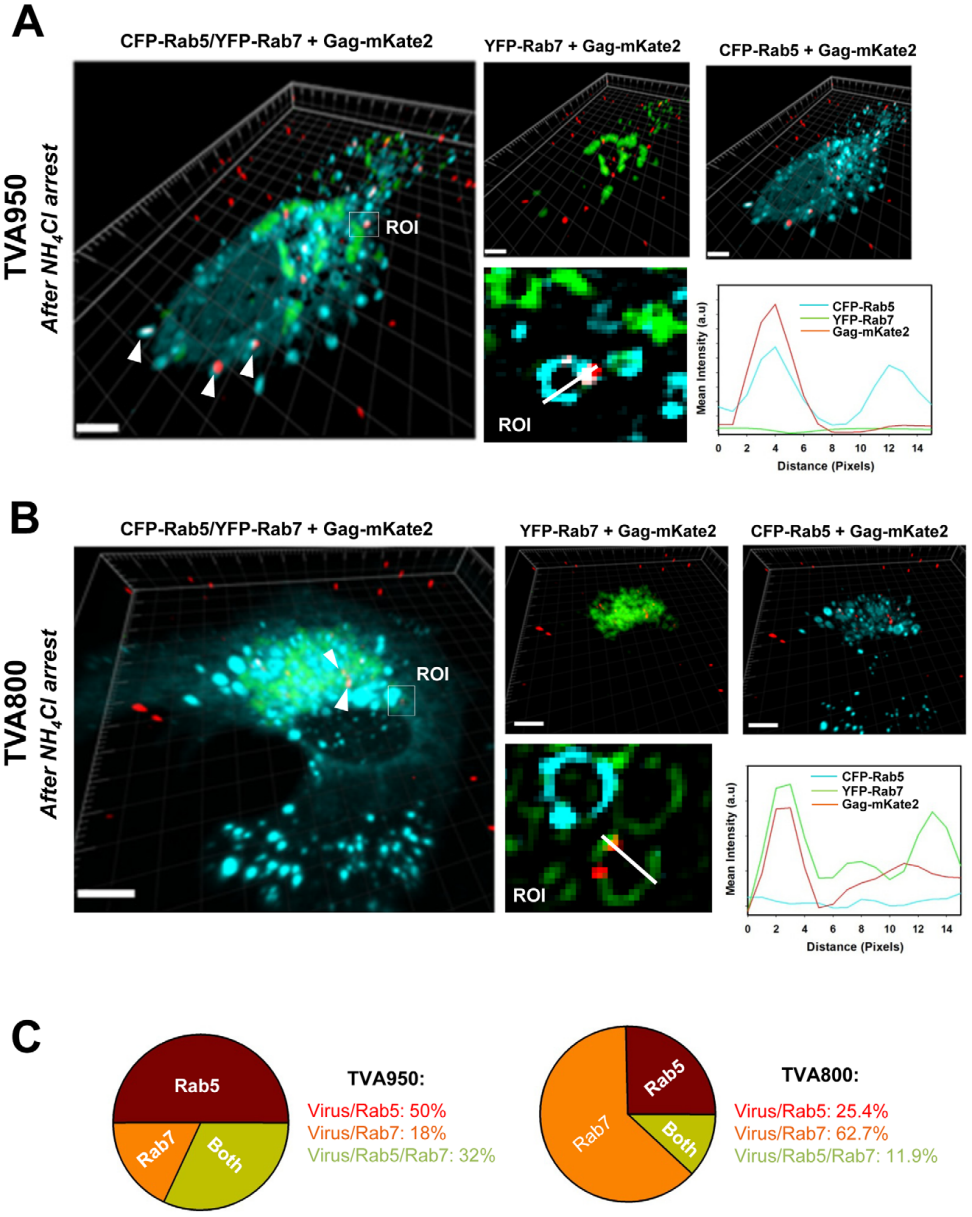
NH_4_Cl-arrested ASLV preferentially resides in Rab5-positive endosomes in TVA950 cells and in Rab7-positive compartments in TVA800 cells. Cells co-transfected with CFP-Rab5 (cyan) and YFP-Rab7 (green) were allowed to internalize ASLV pseudoviruses labeled with Gag-mKate2 (red) for 40 minutes at 37°C in the presence of 70 mM NH_4_Cl. (A) Image projections obtained from 30 confocal slices show viruses in a TVA950 cell expressing CFP-Rab5 and YFP-Rab7. Arrowheads show viral particles co-localized with Rab5-positive puncta. A rectangular region of interest, R0I (10×9 µm) encompassing a viral particle (upper left panel) is enlarged and shown in the lower panel. ROI is a confocal slice corresponding to the maximum intensity in Z for the red channel (Gag-mKate2). (B) Same as in panel A, but for TVA800 cell co-transfected with CFP-Rab5 and YFP-Rab7. Arrowheads show viral particles co-localized with Rab7-positive endosomes. A blow up view of ROI encompassing an internalized virus is shown in the lower panel. Co-localization analysis was done by constructing a line histogram (A and B, lower right panels) for a chosen confocal plane that showed a maximum intensity for all channels (ROIs in A and B). The line histogram shows the changes in fluorescence intensities for all three channels along the white lines (lower middle panels) drawn across the viruses and an endosomal compartment in the ROI (10×9 µm). Local maxima for Rab5 and/or Rab7 coinciding with the maximum of the mKate2 signal were counted as co-localization. Scale bars are 10 µm. (C) Summary of analyses of ASLV pseudovirus co-localization with endosomal markers in TVA950 (left) and TVA800 (right) cells.

To further test the possibility that ASLV is trafficked to different compartments of the two cell lines, we analyzed the virus co-localization with markers of early and late endosomes. Arresting the ASLV fusion by NH_4_Cl greatly simplifies this analysis, since viruses accumulate in distinct intracellular compartments in cells expressing alternative receptor isoforms. Cells were co-transfected with CFP-Rab5 and YFP-Rab7 and allowed to internalize pseudoviruses labeled with Gag-mKate2 (red) for 40 min in the presence of NH_4_Cl. Co-localization analysis revealed that 82% of ASLV pseudoviruses resided in Rab5-positive and mixed Rab5/Rab7-positive compartments in TVA950 cells (Figure 5A, C). In contrast, the majority (63%) of viruses internalized through TVA800 co-localized with Rab7 and only 25% were found in Rab5-positive endosomes (Figure 5B, C).

We also examined the spatial overlap between synchronized ASLV fusion and endosomal markers. ASLV fused with Rab5-positive endosomes of both TVA800 and TVA950 cells (e.g., Figure S8). However, dynamic co-localization analysis of fusion with Rab7-positive compartments could not be reliably performed because of their clustering at the perinuclear space of NH_4_Cl-arrested cells (Figure 5). We therefore chose not to pursue these experiments. Taken together, the above results support the notion that the two receptor isoforms traffic ASLV into distinct intracellular compartments. Since the NH_4_Cl arrest-release protocol leads to efficient infection of cells expressing TVA800 ([16] and data not shown), viral cores must eventually enter into the cytoplasm. The post-fusion steps resulting in capsid liberation into the cytoplasm are addressed below.

### Sub-viral particles released into TVA800 cells reach the cytosol after an additional slow temperature-dependent step

If SVPs are released into endosomal compartments of TVA800 cells, an additional step would be required to deliver these particles into the cytoplasm and establish productive infection (assuming that SVPs represent *bona fide* viral cores). To address this question, we pseudotyped the HIV-1 core carrying the beta-lactamase (BlaM) reporter enzyme with the ASLV Env and used these particles to measure virus-cell fusion [29], [33]. Transfer of the viral core-incorporated BlaM into the cytosol as a result of fusion allows cleavage of a fluorogenic substrate sequestered in the cytoplasm. This substrate is thus inaccessible to the BlaM trapped within the viral particles that fail to fuse.

**Figure 6:**
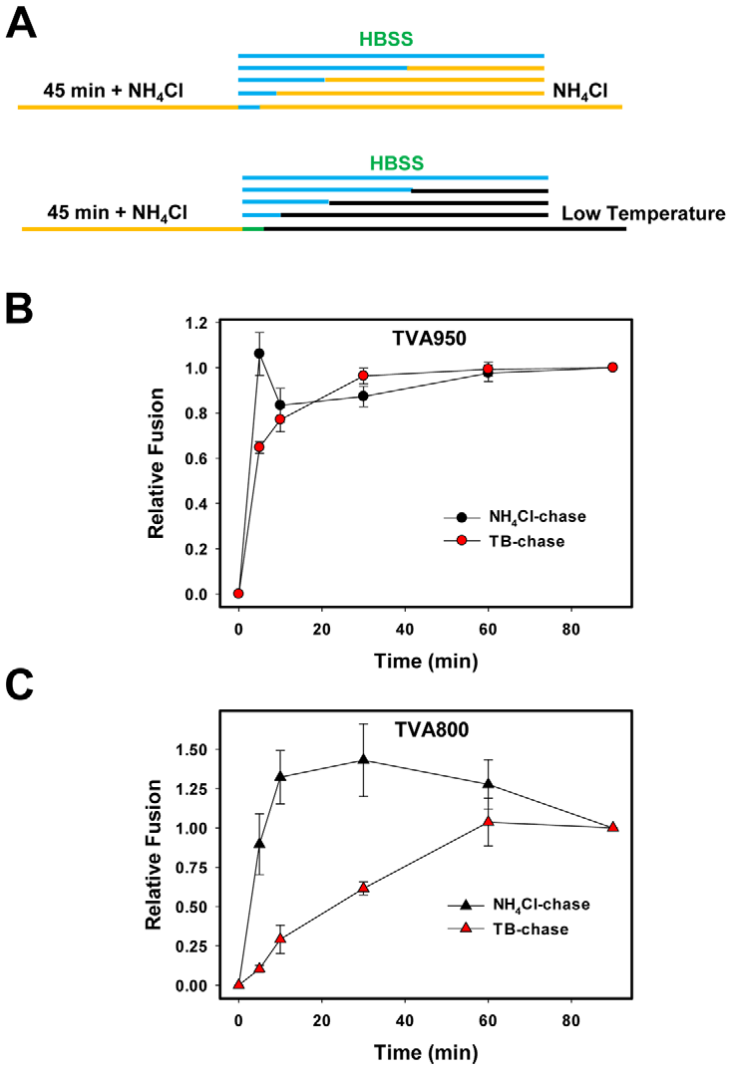
ASLV progression through acid- and temperature-dependent steps of fusion after removal of the NH_4_Cl block. (A) Schematic diagram of the NH_4_Cl arrest/chase experiments. EnvΔCT/BlaM-Vpr pseudoviruses were bound to either TVA950 (B) or TVA800 (C) cells at 4°C. Cells were allowed to internalize virus during 45 min at 37°C in the presence of 70 mM NH_4_Cl (yellow line in A). Fusion was initiated by transferring the cells into HBSS supplemented with 50 µg/ml R99 (blue line in A) for varied times and stopped either by adding back NH_4_Cl containing 50 µg/ml R99 (black circles) or by placing the samples on ice (TB) (red circles). At the end of the chase, cells were chilled on ice, loaded with the BlaM substrate and incubated overnight at 12°C. Data points are means and SEM of combined triplicate measurements from two independent experiments.

ASLV pseudoviruses were internalized by TVA800 or TVA950 cells for 45 min at 37°C in the presence of NH_4_Cl. Fusion was initiated by transferring the cells into HBSS and was stopped after varied times, either by chilling on ice (referred to as the temperature block, TB) or by adding NH_4_Cl to re-neutralize the endosomal pH (Figure 6A). Cells were loaded with the BlaM substrate and incubated overnight at 12°C to allow the substrate cleavage but prevent further fusion. When the NH_4_Cl block was not lifted (t = 0 for the chase experiment), only a background level BlaM signal was detected (Figure 6). Consistent with our imaging results, even a very brief removal of NH_4_Cl allowed fusion to proceed to completion. Adding the NH_4_Cl back after a few minutes no longer reduced the fusion efficiency in either cell line (Figure 6B, C). Similarly, the TB applied even after a brief removal of NH_4_Cl failed to block fusion in TVA950 cells (Figure 6B). In sharp contrast, ASLV escape from low temperature after lifting the NH_4_Cl block was markedly delayed in TVA800 cells (Figure 6C). This delayed resistance to low temperature that occurred with half-time ∼20 min was suggestive of an additional post-fusion step responsible for delivery of viral cores into the cytosol. It is thus conceivable that SVPs are first released into intracellular compartments in NH_4_Cl-arrested TVA800 cells and then enter into the cytosol through an additional Env-independent, temperature-dependent step.

## Discussion

We have previously found that ASLV fuses far less optimally with TVA800 cells compared to TVA950 cells when entering *via* a conventional route [17]. Because these receptor isoforms have identical ectodomains, it is unlikely that their Env binding or priming activities differ considerably. On the other hand, a recent study using cells expressing undetectably low levels of TVA800 and TVA950 arrived to the conclusion that more than one GPI-anchored receptor is required for ASLV infection, whereas a single transmembrane receptor is sufficient for productive entry [34]. However, this finding does not rule out the possibility that post-priming steps of entry determine the ASLV fusion efficiency and thus the minimal number of receptors supporting this process. We favor the model that the higher probability of fusion with TVA950 cells is due to ASLV trafficking to more permissive compartments than those in TVA800 cells [17]. This notion is consistent with data supporting ASLV trafficking to distinct intracellular compartments in cells expressing alternative TVA isoforms [16], [17]. The differences in fusion-permissiveness of distinct endosomes suggest a role for host factors in regulating ASLV fusion and capsid release.

Although NH_4_Cl impairs endosomal trafficking [20]–[22], the arrest/release protocol employed in this study provides an unprecedented opportunity to redirect the ASLV fusion to different compartments and study this process under controlled conditions. This approach permitted: (i) synchronous and uniform acidification of all endosomes harboring the virus; (ii) isolation of low pH-dependent stages of fusion from upstream receptor-priming and virus trafficking; and (iii) enhancement of the viral core release from endosomes. A uniform acid load delivered upon NH_4_Cl removal allowed us to meaningfully compare the fusion-permissiveness of endosomes harboring the virus and to infer their overall topology. In summary, the NH_4_Cl block applied to cells expressing alternative TVA isoforms provided a critical means to direct the ASLV entry through distinct endosomes and gain new insights into early post-fusion processes. We are currently working on visualization of the viral core release upon uninterrupted ASLV entry through alternative TVA isoforms.

**Figure 7:**
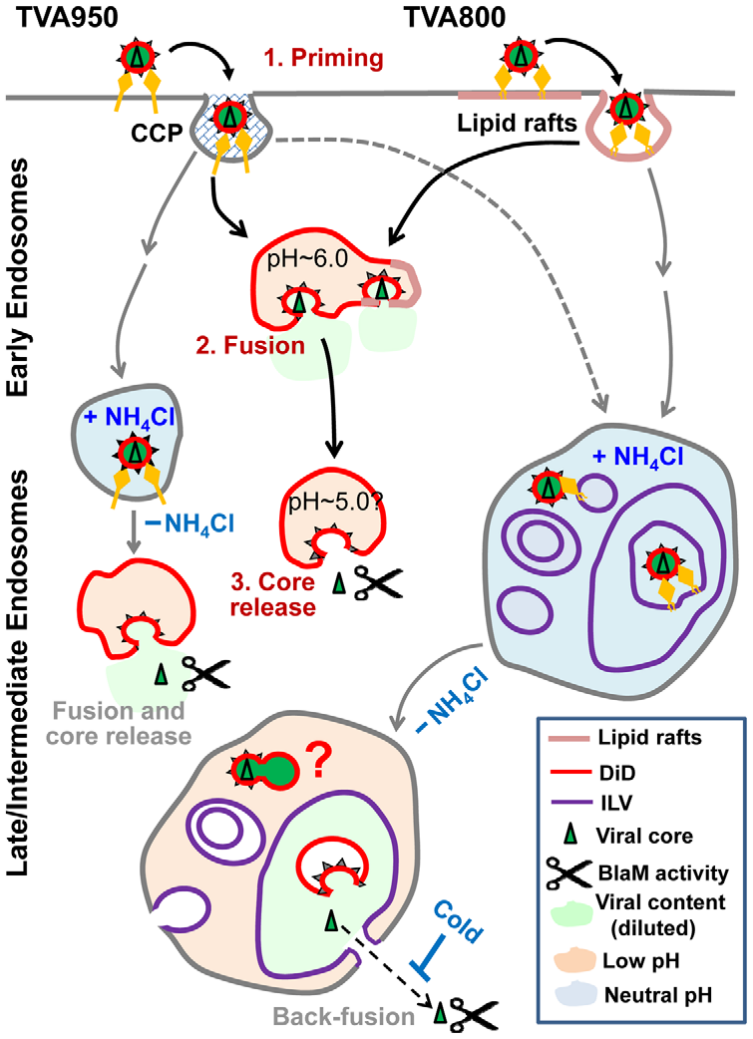
Model for ASLV fusion with cells expressing alternative receptors following the arrest/release protocol. Alternative receptor isoforms direct ASLV entry through distinct pathways that culminate in fusion with early endosomes. Normal entry pathways through TVA800 and TVA950 may converge to early endosomes where mildly acidic pH initiates the viral content release, albeit with different efficiency [17]. In the presence of NH_4_Cl (gray arrows), the virus bypasses early endosomes and enters intermediate compartments of TVA950 cells and late endosome-like compartments of TVA800 cells. Removal of NH_4_Cl initiates fusion with the limiting membrane of intermediate endosomes in TVA950 cells. Subsequent dilation of a fusion pore releases the viral core (triangle) into the cytosol. In TVA800 cells, ASLV is trapped within a small intralumenal vesicle, which is enclosed by a larger vesicle surrounded by the limiting membrane of an endosome. Fusion results in the content and core release into a large intralumenal vesicle. The viral core then traverses two membranes *via* back-fusion of an enlarge vesicle with the limiting membrane of an endosome (dashed arrow). The latter step is likely to be temperature-dependent and independent of ASLV Env.

Novel approaches introduced in this study enabled probing the sites of ASLV fusion. As a rule, sub-viral particles liberated in NH_4_Cl-arrested TVA950 cells sensed the cytosolic pH, consistent with the ASLV fusion with the limiting membrane of an endosome (Figure 7). In contrast, cores released into TVA800 cells experienced the pH responses characteristic of endosomal compartments lacking the active buffering ability of the cytosol. Subsequent core release into the cytosol was detected by the BlaM assay (Figure 6). The nature of this slow temperature-dependent post-fusion step is presently unknown. We propose the following two-step model for ASLV fusion with TVA800 cells following the NH_4_Cl arrest/release protocol.

Since the viral membrane marker does not spread over a larger area as a result of fusion, it is likely that ASLV fuses with a small intralumenal vesicle. However, to account for disappearance of the viral content marker through a marked dilution for increased mobility of the released core, virus has to reside within a small vesicle (Figure 7). We propose that a small virus-carrying vesicle is in turn enclosed by a large intralumenal vesicle within an endosome. Multilamellar endosomes have been observed under physiological conditions and in the presence of NH_4_Cl [21], [35]–[37]. This triple-vesicle enclosure is based on several considerations. First, the interior of an intralumenal vesicle is topologically equivalent to the cytoplasm, so that internalized virus cannot reside within that vesicle. Second, core liberation into the cytosol from the remaining double membrane enclosure can occur through back-fusion [5] (Figure 7), consistent with a slow temperature-dependent step following the low pH-mediated fusion with TVA800 cells (Figure 6).

The proposed ASLV entry pathway is somewhat similar to that postulated for the Vesicular Stomatitis Virus (VSV). VSV is thought to fuse “laterally” with an intralumenal vesicle (Figure 7, question mark) and release its capsid through back-fusion with the limiting membrane of a late endosome [5] (but see [38] for an opposite view). Our experimental data are difficult to reconcile with the VSV model. First, as pointed out above, fusion with small intralumenal vesicles should not result in sufficient dilution of the viral content to account for disappearance of the GFP signal (Figure 7). Second, the capsid release into a small vesicle will not allow detectable spatial separation from the viral envelope. Third, back-fusion of virus/vesicle hybrids should spill the viral membrane marker into a large area of the endosome's limiting membrane, an event that has been observed only once in our experiments (data not shown). We therefore favor the “triple-enclosure” model presented above. It would be interesting to elucidate trafficking processes that entrapped the virus within such structure.

Results presented in this study revealed the previously unappreciated aspects of viral entry through distinct pathways. Synchronous triggering and real-time visualization of ASLV fusion in cells expressing alternative receptors demonstrates that the identity of virus-harboring compartments determines the efficiency of fusion. Furthermore, the viral core release in NH_4_Cl-arrested TVA800 cells proceeds through a complex pathway that involves sequential pH-dependent and temperature-dependent steps. The latter post-fusion step(s) is unlikely to be mediated by ASLV Env. This provides strong evidence for the existence of back-fusion, which has been implicated in entry of several viruses [5], [7] and of the anthrax toxin lethal factor [39]. Novel tools developed in this study can be used for probing the sites of fusion and capsid release of other viruses and to gain insights into vesicular trafficking and cargo sorting processes.

## Materials and Methods

### Cell lines, viruses and reagents

African green monkey kidney CV-1 and human embryonic kidney HEK293T/17 cell lines were obtained from ATCC (Manassas, VA). HeLa-derived indicator TZM-bl cells expressing CD4, CXCR4 and CCR5 (donated by Drs. J.C. Kappes and X. Wu [40] were obtained from the NIH AIDS Research and Reference Reagent Program. CV-1-derived CV-1/TVA800 and CV-1/TVA950 cells expressing high levels of alternative TVA receptors have been described previously [17]. TZM-bl cells expressing TVA800 or TVA950 were obtained by transduction with VSV-G pseudotyped retroviral vectors pCMMP-TVA950 or pCMMP-TVA800, as described previously [16]. Cells expressing high levels of either TVA receptor were sorted by flow cytometry using a FACS Aria II (BD Biosciences, San Jose, CA) after binding to the subgroup A ASLV SU-IgG fusion protein [16] and a goat anti-rabbit FITC-conjugated secondary antibody (Sigma, St. Louis, MO). CV-1-derived cells were grown in Dulbecco's modified Eagle high glucose medium (DMEM, Cellgro, Manassas, VA) supplemented with 10% Cosmic Calf Serum (HyClone Laboratories, Logan, UT) and 100 U penicillin-streptomycin (Gemini Bio-Products, West Sacramento, CA). TZM-bl cells were grown in high glucose DMEM supplemented with 10% Fetal Bovine Serum (FBS, HyClone Laboratories, Logan, UT) and 100 U penicillin-streptomycin. HEK 293T/17 cells were maintained in high glucose DMEM supplemented with 10% FBS, 100 U penicillin/streptomycin and 0.5 mg/ml G418 sulfate.

The ASLV (subgroup A) Env-derived R99 peptide (∼95% purity by HPLC) was synthesized by Macromolecular Resources (Fort Collins, CO). The lipophilic dye DiD (1,1′-dioctadecyl-3,3,3′,3′-tetramethylindodicarbocyanine, 4-chlorobenzenesulfonate salt), Hoechst-33342 nuclear stain and pHrodo dextran were purchased from Invitrogen (Carlsbad, CA). Ammonium chloride was purchased from Sigma. Hank's balanced salt solution with calcium and magnesium without phenol red (HBSS) and Phosphate-Buffered saline (PBS) were from Cellgro. Sodium pyruvate was from Hyclone.

The subgroup A ASLV Env glycoprotein lacking the cytoplasmic domain (designated EnvΔCT) has been described previously [41]. The HIV-1 based packaging vector pR8ΔEnv lacking the *env* gene was from Dr. D. Trono (University of Geneva, Switzerland). The pMM310 vector expressing BlaM-Vpr (donated by Dr. M. Miller [42]) was obtained from the NIH AIDS Research and Reference Reagent Program. Vectors expressing MLV Gag-pol, Gag-GFP, MLV LTR lacZ, pECFP-C1-Rab5 and pEYFP-C1-Rab7 [43] were a gift from Dr. W. Mothes (Yale University). The MLV Gag-mKO expressing vector has been described previously [24]. The mRFP-Rab5 and mRFP-Rab7 expression vectors were obtained from Addgene (Cambridge, MA).

### Construction of MLV Gag-mKate2 and GFP-ICAM-1 markers

The construction of GFP-ICAM-1 was done as follows. The eGFP gene was amplified using KOD Xtreme DNA polymerase (Novagen) and the following primers: 5′-T*AAGCTT*CTCGAG GTGAGCAAGGGCGAGGAGCTGTTC-3′ (forward) and 5′-T*GAATTC*TT CTTGTACAGCTCGTCCATGCCGAGAGTG-3′ (reverse). The amplified fragment was cloned into pCR4blunt-topo vectors using TOPO cloning kit (Invitrogen). After the verification of the sequence of the gene of interest, the EcpH sequence in the EcpH-ICAM-1 vector [29] was replaced with the eGFP fragment using *HindIII* and *EcoRI* restriction sites (italicized regions in the primer sequences). The pMLV-Gag-mKate2 plasmid was constructed by amplifying the mKate2 gene by PCR using KOD Xtreme DNA polymerase (Novagen) and the following forward and reverse primers: 5′-ATTGC*GGATCC*GGCGGCGGTGGA*GCTAGC*GTGAGCGAGCTGATTAAGGAGAAC-3′ and 5′-TTCC*GCCGGC*TTA*GATATC*TCTGTGCCCCAGTTTGCTAGGGAG-3′, which contained the *BamHI* and *NaeI* restriction cleavage sites, respectively. The amplified fragment was first clone into the pCR4blunt-topo vector, using TOPO cloning kit (Invitrogen), and its sequence was verified. The YFP gene in pMLV-Gag-YFP vector [44] was replaced with the mKate2 sequence in pCR4blunt-topo by restriction digestion with *BamHI* (New England Biolab) and *NaeI* (New England Biolab) and ligation with T4 DNA ligase (New England Biolab).

### Virus production and transfections

Fluorescently labeled pseudoviruses were produced in HEK293T/17 cells using PolyFect Transfection reagent (Qiagen, Valencia, CA). Cells grown on a 10 cm dish were transfected with 2 µg MLV-Gag-Pol, 1 µg MLV-Gag-GFP or MLV-Gag-mKO, 3 µg pMLV-LTR-LacZ and 3 µg of the cytoplasmic tail-truncated ASLV-A Env (EnvΔCT). For ASLV Env-pseudotyped virus carrying a membrane-incorporated GFP, 2 µg GFP-ICAM-1 were added to the transfection DNA mixture. Twenty four hours post-transfection, cells were labeled with 10 µM DiD in Opti-MEM (Invitrogen) for 4 h in the CO_2_ incubator at 37°C, as described in [41], washed, covered with 6 ml of fresh phenol red-free DMEM/10% FBS, and incubated for an additional 24 h. Virus-containing medium was collected 48 h post-transfection, passed through a 0.45 µM filter, aliquoted and stored at −80°C. The infectious titer was determined by a β-Gal assay in CV-1 cells expressing TVA800, as described previously [29], [45]. To produce pseudoviruses containing the β-lactamase-Vpr (BlaM-Vpr), a 10 cm dish of HEK293T/17 cells was transfected with 2 µg pR8ΔEnv, 2 µg pMM310 vector expressing BlaM-Vpr, 1 µg pcRev, and 3 µg EnvΔCT expression vector, using PolyFect, as described above. The infectious titer was determined by β-Gal assay in TZM-bl cells expressing TVA950.

For transient expression of endosomal markers, CV-1 cells expressing TVA800 or TVA950 were grown to 80% confluency on glass-bottom 35 mm Petri dishes (Mattek, Ashland, Massachusetts) in phenol red-free DMEM. Cells were transfected with 0.5 µg of CFP-Rab5 and 0.5 µg YFP-Rab7, using Nanofectin transfection reagent (PAA Laboratories, Dartmouth, MA), and used for imaging 24 h post-transfection.

### Virus-cell fusion assay

EnvΔCT-pseudotyped pseudoviruses bearing the BlaM-Vpr chimera were bound to CV-1 cells expressing TVA800 or TVA950 in 96-well stripwell plates (Corning, NY) by centrifugation at 2095×g, 4°C for 30 min (MOI = 1). Unbound virus was washed off with ice-cold HBSS, and cells were incubated in isotonic HBSS/2% FBS (pH 7.8) containing 70 mM NH_4_Cl were incubated for 45 min at 37°C. Fusion of internalized viruses trapped in endosomes was initiated by transferring the cells into HBSS/2% FBS supplemented with 50 µg/ml of Env-derived R99 peptide (to prevent fusion of not internalized viruses) for varied times and stopped either by chilling the cells on ice (referred to as the temperature block, TB) or by adding 70 mM NH_4_Cl containing 50 µg/ml R99. The medium was removed from wells, and cells were loaded with fluorescent CCF4-AM substrate (Invitrogen) and incubated overnight at 12°C.

### Imaging of single virus fusion

Unless stated otherwise, imaging experiments were performed using the Personal DeltaVision imaging system (Applied Precision, Issaquah, WA) equipped with an environmental enclosure that maintained the samples at 37°C and high relative humidity. Double- (Gag-GFP/DiD) and triple-labeled (Gag-mKO/GFP-ICAM/DiD) viruses and cellular proteins tagged with a monomeric red fluorescent protein (mRFP-Rab5 and mRFP-Rab7) were imaged using an UPlanFluo 40×/1.3 NA oil objective (Olympus) and a standard DAPI/FITC/TRITC/Cy5 filter set (Chroma, Bellows Falls, VT). The fluorescence emission was collected by an EM-CCD camera (Photometrics). For double-labeled viruses, two consecutive images were collected every 1.5 sec for up to 10 min. Triple-labeled viruses were imaged every 2.2 seconds for the same period of time. In order to compensate for the axial drift during acquisition, we used the *UltimateFocus* feature (Applied Precision), which automatically compensates for changes in the coverslip position.

CV-1 cells expressing either TVA950 or TVA800 receptor were grown to near confluency on glass-bottom 35 mm Petri dishes (Mattek) in phenol red-free growth medium. Cells were placed on ice, washed with cold HBSS, and centrifuged with ∼1.5·10^4^ IU of viruses in 100 µl HBSS/2% FBS at 2,100×g (4°C) for 20 min. Unbound viruses were removed by washing with cold HBSS, and cells were incubated in isotonic HBSS/2% FBS supplemented with 70 mM NH_4_Cl (pH = 7.8) for 40 min at 37°C to allow virus uptake. For imaging, cells were transferred into serum-free HBSS containing 70 mM NH_4_Cl and mounted on the microscope stage maintained at 37°C. Solutions around the cells in an image field were changed using a 4-channel miniature perfusion system (Bioscience Tools, San Diego, California) controlled through the Worx imaging software. Solutions were applied locally through a 100 µm plastic tip positioned close to the imaged cells, as described in [29]. The following two pre-warmed solutions were applied to cells: HBSS supplemented with 70 mM NH_4_Cl (pH = 7.8) and plain HBSS (pH = 7.2). At the end of imaging experiments, cells were returned to the NH_4_Cl-containing buffer.

### Image analysis

Spot-enhancing filter 2D plugin from ImageJ [46] was applied to background-subtracted images to improve the signal to noise ratio. Virus tracking was performed with the 64-bit software module from Imaris (BitPlane, Zurich, Switzerland), using an auto-regressive algorithm. Tracking provided quantitative information regarding the mean fluorescence intensities of the viral content and membrane markers, particle's instantaneous velocity, trajectory and the mean square displacements (MSD). The two-color kymographs were obtained, using the Volume Viewer plugin from ImageJ.

The generalized two-dimensional diffusion coefficients (D) were obtained from the y-axis intercepts (y_0_) for the MSD logarithmic plot using the equation, as described in [47]:
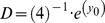
(1)Only the linear part of MSD following the core release was used to calculate D. The linear MSD regime typically ranged from 10 to 25 seconds after core release. For co-localization analysis, images were acquired after 40 min incubation with 70 mM NH_4_Cl at 37°C (unless stated otherwise), using the Zeiss LSM780 microscope (Carl Zeiss Microscopy), and a 63×/1.2 NA oil immersion objective. Between 25 and 30 confocal Z sections were acquired (0.3 µm apart) using a pinhole set at 1 Airy unit. Spectral unmixing to correct for bleed-through between CFP-Rab5/YFP-Rab7 and 3D image reconstruction were carried out with the Zen and Imaris software, respectively. Image analysis for co-localization was performed after subtracting the background, selecting a meaningful range of apparent particle sizes (between 0.5 and 3 µm) and comparing the line histograms of CFP-Rab5, YFP-Rab7 and Gag-mKate2 intensity distributions. A viral particle was considered to be co-localized with an endosomal marker, if the respective peaks of line histograms coincided (i.e., an endosomal marker exhibited a local fluorescence maximum coinciding with that of a viral particle).

## Supporting Information

Figure S1 **Measurements of endosomal pH using triple-labeled pseudoviruses.** (A) Changes in pH upon addition/removal of NH_4_Cl. NH_4_^+^ permeates through a lipid membrane in its neutral ammonia form, which then acquires a proton and raises the pH of the target compartment. Conversely, NH_4_^+^ efflux leaves excess protons and thus lowers the pH. (B) Distributions of endosomal pH in CV-1 cells expressing TVA950. Cells were pre-loaded with 40 µg/ml of pHrodo dextran according to the manufacturer's instructions, and fluorescence emission at 585 nm was recorded. The fluorescence of pHrodo dextran increases at low pH [23], so acidic compartments are brighter than neutral endosomes. Total fluorescence intensity of single endosomes was measured and normalized to the maximum fluorescence intensity in control (untreated) cells. Graph shows the normalized distribution of fluorescence intensities of endosomes in untreated TVA950 cells (green bars), in the presence of 70 mM NH_4_Cl (following a 40 min-incubation at 37°C, dark cyan bar), and immediately after replacing NH_4_Cl with HBSS (NH_4_Cl/HBSS, blue bars). (C) Changes in the average fluorescence of five pHrodo dextran-laden endosomes in CV-1 cells upon perfusion with 70 mM NH_4_Cl in HBSS (white horizontal bar) with an intermittent perfusion with plain HBSS for 2 min (black horizontal bar). (D) Pseudoparticles carrying ASLV-A Env were co-labeled with MLV Gag-mKO (core), GFP-ICAM-1 (membrane) and DiD (membrane). Viruses were allowed to adhere to poly-L-lysine coated coverslips and imaged at room temperature in buffers adjusted to different pH values. Only particles positive for all three markers, Gag-mKO (blue), GFP-ICAM-1 (green) and DiD (red) where used for analysis (exemplified by two encircled white particles on the top right panel. The pH 5.2 citrate buffer strongly reduces the GFP-ICAM-1 fluorescence (lower panel) without affecting the reference DiD signal or Gag-mKO fluorescence. (E) Changes in the GFP fluorescence in response to citrate buffers of different acidity. Buffers were applied to coverslip-immobilized viruses through a local perfusion, starting with the pH 7.0 HBSS, followed by a citrate buffer adjusted to an indicated pH between 4.6 and 6.3. Samples were perfused with neutral HBSS at the end of experiment. The experimental data were smoothed using a running average algorithm. (F) The pH calibration curve obtained by plotting the average ratio of GFP-ICAM-1 and DiD signals from 10 viruses as a function of pH. The last 5 time points of the GFP and DiD signals in panel E before returning to a neutral buffer were used for the calibration curve.
(TIF)

Figure S2 **Dynamics of endosomal, cytosolic and intraviral pH upon removal of NH_4_Cl.** (A) The average intensity profiles for non-fusogenic ASLV pseudoviruses labeled with GFP-ICAM-1, DiD and MLV Gag-mKO (pooled data using cells expressing TVA800 and TVA950). Cells were perfused with NH_4_Cl (white horizontal bar) followed by a 2 min-perfusion with HBSS (black bar). The changes in average fluorescence ratio of GFP over DiD of 20 randomly chosen particles following the NH_4_Cl removal/addition are shown (green circles). The average pH trace (pink line) was obtained from the calibration curve shown in Figure S1F. Error bars correspond to the standard deviation from the 20 GFP/DiD ratios was obtained for each time point. (B) TVA800 and TVA950 cells transiently expressing cytosolic eGFP were stained with DiD and imaged. The average ratio of GFP/DiD (green dots) was obtained for selected regions of interest within different cells. The corresponding error was obtained by calculating the standard deviation of each time point for every GFP/DiD measurement. The corresponding average pH trace (pink line) was obtained from the calibration curve shown in Figure S1F. The resting cytosolic pH value was set to 7.3, as shown in [25]. (C) Average decay in the intraviral Gag-GFP (plotted as Gag-GFP/DiD ratio) caused by removal of NH_4_Cl. Each datum point is obtained by averaging fluorescence ratios from 20 randomly chosen viral particles. The corresponding pH trace (pink line) was obtained from the calibration curve shown in Figure 1F.
(TIF)

Figure S3 **Analysis of releasable Gag-GFP pool in ASLV pseudoviruses.** (A) ASLV pseudoviruses co-labeled with Gag-GFP/DiD were immobilized on coverslips and treated with 0.1 mM saponin at room temperature. Images were taken before and 2–3 min after exposure to saponin, which mediated a quick loss of green fluorescence puncta without noticeably affecting the DiD fluorescence. (B) Analysis for the particles before and after saponin treatment showed that 30% were completely resistant to lysis, 23% lost only a portion of their fluorescence, and 47% completely lost the GFP marker. The partial release phenotype was unlikely due to virus aggregation, since larger particles were excluded from analysis.
(TIF)

Figure S4 **Incomplete separation of GFP- and DiD-labeled puncta during the NH_4_Cl arrest/release protocol.** ASLV pseudoviruses co-labeled with Gag-GFP (green) and DiD (red) were subjected to the NH_4_Cl arrest/release protocol in TVA950 cells (see Materials and Methods for details). (A) In spite of the lack of full separation of green and red puncta (image panels), the GFP fluorescence changes (green dots) during both HBSS perfusion intervals paralleled the changes in the cytosolic pH (pink lines) under the same perfusion conditions. The right panel shows the trajectories of the SVP (green) and an endosome (red). The initial, fully overlapping part of the trajectory is colored blue. (B) Line histograms showing the spatial overlap of GFP-Gag (green line) and DiD (red) puncta before (t = 0, left) and after (t = 140 sec, right) partial SVP separation from an endosome.
(TIF)

Figure S5 **Examples of SVP release (single HBSS pulse).** Two examples of spatial separation of GFP- and DiD-tagged puncta following the NH_4_Cl arrest/release protocol for TVA800 cells (A) and TVA950 cells (B). In all cases, SVP release occurred during HBSS perfusion (blue asterisk) and is detected by the abrupt drop in the DiD intensity signal (red circles). The cytosolic pH profile (pink lines in all panels) is also shown. In the case of TVA800 cells (A), the GFP fluorescence recovery occurs only after returning to NH_4_Cl (t = 180 sec), a behavior expected for endosomal compartments. In TVA950 cells the change in GFP-Gag intensity after SVP release paralleled that of cytosolic pH, suggesting that the viral capsid was released into the cytoplasm. The trajectories for the GFP-tagged SPVs (solid green line) together with the DiD-recipient vesicle trajectories (solid red line) are shown below each intensity profile. In all cases, the motilities of SVPs after their separation from endosomes were different from those of endosomes that received DiD. Clearly, the SPV movement is more restricted in TVA800 cells (A) than in TVA950 cells (B). Diffusion coefficients (calculated as described in Materials and Methods) shown next to the trajectories are 2-fold greater for SPVs tracked in TVA950 cells compared to TVA800 cells.
(TIF)

Figure S6 **Examples of spatial SVP release (double HBSS pulse).** Examples of delayed spatial separation of GFP- and DiD-tagged puncta following the NH_4_Cl arrest/release protocol in TVA800 (A) and TVA950 cells (B). SVPs were released after the first HBSS perfusion pulse, which ended at t = 180 sec. Therefore a second HBSS pulse was applied (between 240 and 360 sec) to assess the localization of the released cores. This protocol revealed different pH environment for the released capsids in cells expressing alternative TVA receptors. The pH profile of the cytosol (solid pink line in all panels) shows a fast recovery following the first and the second HBSS pulses. This behavior is matched by the GFP intensity profiles from SPVs (green dots) released into TVA950 cells (B), but not into TVA800 cells (A). The trajectories for the GFP-Gag (solid green line) signal together with the DiD (solid red line) are also shown under each intensity profile. Comparison of the SVP trajectories in TVA800 and TVA950 cells shows that their mobility was more restricted in TVA800 cells (see also Figure 3 and Figure S5). The diffusion coefficients calculated as described in the Materials and Methods are also given for the representative particles.
(TIF)

Figure S7 **Example of core release into an endosome of a TVA950 cell.** (A) Spatial separation of GFP- and DiD-tagged puncta in a TVA950 cell occurred during HBSS perfusion (blue asterisk) as evidenced by the abrupt drop in the DiD intensity (red circles). The GFP fluorescence recovers only after returning to NH_4_Cl (t = 180 sec), suggesting the SVP release into an endosomal compartment. The endosome and SVP trajectories are shown in B. The mean square displacement of SVP and DiD are also depicted in C. The linear part of the MSD after SVP release (between dashed lines in C) was chosen to generate a log-log plot for the MSD over time (D). The diffusion coefficient (D = 0.16 µm^2^/sec) of SVP was calculated using the y_0_ value determined from the intercept of a linear fit with the Y axis and applying Eq. 1 (see the main text for details).
(TIF)

Figure S8 **Single ASLV fusion with Rab5-positive endosome.** TVA950 cells were transiently transfected with a marker for early endosomes, mRFP-Rab5 (A). ALSV pseudoviruses co-labeled with Gag-GFP (green) and DiD (blue) were internalized by cells in the presence of NH_4_Cl for 40 min at 37°C. Cells were initially perfused with 70 mM NH_4_Cl in HBSS followed by perfusion with plain HBSS for 2 min (black horizontal bars) and, finally, with NH_4_Cl. (A, B) Fusion with a Rab5-positive endosome in TVA950 cell is illustrated. Double-labeled viruses co-localized with mRFP-Rab5 endosomes (red) appear white. The white square in (A), delineates a region of interest magnified in (B) and shown for different time points corresponding to perfusion with different buffers t = 0 sec (NH_4_Cl), t = 90 sec (HBSS) and t = 250 sec (NH_4_Cl). The arrowheads in the merged panels mark two particles undergoing fusion in Rab5-positive endosomes. (C) Fusion was associated with a complete loss of GFP fluorescence (green circles) from virions during the HBSS perfusion, while the fluorescence intensities of DiD (blue circles) and mRFP-Rab5 (red circles) remained steady. The corresponding two-color kymograph (DiD/Gag-GFP) and three-color kymograph (DiD/Gag-GFP and mRFP-Rab5) are also shown. For comparison, the intensity profile for a non-fusogenic particle within a Rab5-positive endosome is shown in panel D along with the corresponding two-color kymograph (DiD/Gag-GFP).
(TIF)

Video S1 **Failure of internalized ASLV pseudovirus to fuse upon HBSS perfusion is associated with reversible quenching of fluorescence of the GFP-tagged viral content marker.** An ASLV pseudovirus co-labeled with Gag-GFP and DiD was internalized by a CV-1/TVA950 cell in the presence of NH_4_Cl. Cells were sequentially perfused with 70 mM NH_4_Cl in HBSS followed by perfusion with plain HBSS for 2 min (as indicated in the upper left corner) and, finally, with NH_4_Cl. The GFP signal from virion fully recovered after returning to NH_4_Cl. The movie is played at 5 frames/sec.
(AVI)

Video S2 **Synchronized fusion of an ASLV pseudovirus with an endosome.** A pseudovirus co-labeled with Gag-GFP and DiD was internalized by a CV-1/TVA950 cell in the presence of NH_4_Cl. Cells were initially perfused with 70 mM NH_4_Cl in HBSS (as indicated at the left corner of the field) followed by perfusion with plain HBSS for 2 min (white thick horizontal letters at the left corner of the video) and, once more, with NH_4_Cl. Virus-endosome fusion is initiated during the HBSS perfusion, which results in acidification of endosomal lumen. Virus-endosome fusion is manifested in irreversible loss of the GFP-tagged content marker. The movie is played at 5 frames/sec.
(AVI)

Video S3 **Spatial separation of GFP- and DiD-tagged puncta in a TVA950 cell following the NH_4_Cl arrest/release protocol.** A co-labeled particle split into two during HBSS perfusion (white circle), and the green and red sub-particles continued to drift apart (thick lines indicating trajectories). The fluorescence profile shown in Figure 2B was obtained for the particle shown in this movie. The movie is played at 5 frames/sec.
(AVI)

Video S4 **Spatial separation of GFP- and DiD-tagged puncta in a TVA800 cell following the NH_4_Cl arrest/release protocol.** A co-labeled particle undergoes split into two during HBSS perfusion (white circle), the restricted mobility and change in intensity after changing HBSS to NH_4_Cl is apparent. The fluorescence profile shown in Figure 2C was obtained for the particle shown in this movie. The movie is played at 5 frames/sec.
(AVI)

## References

[1] Mercer J, Schelhaas M, Helenius A (2010). Virus entry by endocytosis.. Annu Rev Biochem.

[2] Harrison SC (2008). Viral membrane fusion.. Nat Struct Mol Biol.

[3] Smith AE, Helenius A (2004). How viruses enter animal cells.. Science.

[4] White JM, Delos SE, Brecher M, Schornberg K (2008). Structures and mechanisms of viral membrane fusion proteins: multiple variations on a common theme.. Crit Rev Biochem Mol Biol.

[5] Le Blanc I, Luyet PP, Pons V, Ferguson C, Emans N (2005). Endosome-to-cytosol transport of viral nucleocapsids.. Nat Cell Biol.

[6] Zaitseva E, Yang S-T, Melikov K, Pourmal S, Chernomordik LV (2010). Dengue Virus Ensures Its Fusion in Late Endosomes Using Compartment-Specific Lipids.. PLoS Pathog.

[7] Pasqual G, Rojek JM, Masin M, Chatton JY, Kunz S (2011). Old world arenaviruses enter the host cell via the multivesicular body and depend on the endosomal sorting complex required for transport.. PLoS Pathog.

[8] Chandran K, Sullivan NJ, Felbor U, Whelan SP, Cunningham JM (2005). Endosomal proteolysis of the Ebola virus glycoprotein is necessary for infection.. Science.

[9] Schornberg K, Matsuyama S, Kabsch K, Delos S, Bouton A (2006). Role of endosomal cathepsins in entry mediated by the Ebola virus glycoprotein.. J Virol.

[10] Barnard RJ, Narayan S, Dornadula G, Miller MD, Young JA (2004). Low pH is required for avian sarcoma and leukosis virus Env-dependent viral penetration into the cytosol and not for viral uncoating.. J Virol.

[11] Mothes W, Boerger AL, Narayan S, Cunningham JM, Young JA (2000). Retroviral entry mediated by receptor priming and low pH triggering of an envelope glycoprotein.. Cell.

[12] Melikyan GB, Barnard RJ, Markosyan RM, Young JA, Cohen FS (2004). Low pH Is Required for Avian Sarcoma and Leukosis Virus Env-Induced Hemifusion and Fusion Pore Formation but Not for Pore Growth.. J Virol.

[13] Elleder D, Melder DC, Trejbalova K, Svoboda J, Federspiel MJ (2004). Two different molecular defects in the Tva receptor gene explain the resistance of two tvar lines of chickens to infection by subgroup A avian sarcoma and leukosis viruses.. J Virol.

[14] Bates P, Young JA, Varmus HE (1993). A receptor for subgroup A Rous sarcoma virus is related to the low density lipoprotein receptor.. Cell.

[15] Young JA, Bates P, Varmus HE (1993). Isolation of a chicken gene that confers susceptibility to infection by subgroup A avian leukosis and sarcoma viruses.. J Virol.

[16] Narayan S, Barnard RJ, Young JA (2003). Two retroviral entry pathways distinguished by lipid raft association of the viral receptor and differences in viral infectivity.. J Virol.

[17] Jha NK, Latinovic O, Martin E, Novitskiy G, Marin M (2011). Imaging single retrovirus entry through alternative receptor isoforms and intermediates of virus-endosome fusion.. PLoS Pathog.

[18] Narayan S, Young JA (2004). Reconstitution of retroviral fusion and uncoating in a cell-free system.. Proc Natl Acad Sci U S A.

[19] Moolenaar WH, Tsien RY, van der Saag PT, de Laat SW (1983). Na+/H+ exchange and cytoplasmic pH in the action of growth factors in human fibroblasts.. Nature.

[20] Grant KI, Casciola LA, Coetzee GA, Sanan DA, Gevers W (1990). Ammonium chloride causes reversible inhibition of low density lipoprotein receptor recycling and accelerates receptor degradation.. J Biol Chem.

[21] Engel S, Heger T, Mancini R, Herzog F, Kartenbeck J (2011). Role of endosomes in simian virus 40 entry and infection.. J Virol.

[22] Huotari J, Helenius A (2011). Endosome maturation.. EMBO J.

[23] Ogawa M, Kosaka N, Regino CAS, Mitsunaga M, Choyke PL (2010). High sensitivity detection of cancer in vivo using a dual-controlled activation fluorescent imaging probe based on H-dimer formation and pH activation.. Mol Biosyst.

[24] de la Vega M, Marin M, Kondo N, Miyauchi K, Kim Y (2011). Inhibition of HIV-1 endocytosis allows lipid mixing at the plasma membrane, but not complete fusion.. Retrovirology.

[25] Llopis J, McCaffery JM, Miyawaki A, Farquhar MG, Tsien RY (1998). Measurement of cytosolic, mitochondrial, and Golgi pH in single living cells with green fluorescent proteins.. Proc Natl Acad Sci U S A.

[26] Goldsmith DJ, Hilton PJ (1992). Relationship between intracellular proton buffering capacity and intracellular pH.. Kidney Int.

[27] Obara M, Szeliga M, Albrecht J (2008). Regulation of pH in the mammalian central nervous system under normal and pathological conditions: facts and hypotheses.. Neurochem Int.

[28] Markosyan RM, Cohen FS, Melikyan GB (2005). Time-resolved imaging of HIV-1 Env-mediated lipid and content mixing between a single virion and cell membrane.. Mol Biol Cell.

[29] Miyauchi K, Kim Y, Latinovic O, Morozov V, Melikyan GB (2009). HIV enters cells via endocytosis and dynamin-dependent fusion with endosomes.. Cell.

[30] Mire CE, White JM, Whitt MA (2010). A spatio-temporal analysis of matrix protein and nucleocapsid trafficking during vesicular stomatitis virus uncoating.. PLoS Pathog.

[31] Qian H, Sheetz MP, Elson EL (1991). Single particle tracking. Analysis of diffusion and flow in two-dimensional systems.. Biophys J.

[32] Manneville JB, Etienne-Manneville S, Skehel P, Carter T, Ogden D (2003). Interaction of the actin cytoskeleton with microtubules regulates secretory organelle movement near the plasma membrane in human endothelial cells.. J Cell Sci.

[33] Cavrois M, De Noronha C, Greene WC (2002). A sensitive and specific enzyme-based assay detecting HIV-1 virion fusion in primary T lymphocytes.. Nat Biotechnol.

[34] Gray ER, Illingworth CJ, Coffin JM, Stoye JP (2011). Binding of more than one Tva800 molecule is required for ASLV-A entry.. Retrovirology.

[35] Futter CE, Pearse A, Hewlett LJ, Hopkins CR (1996). Multivesicular endosomes containing internalized EGF-EGF receptor complexes mature and then fuse directly with lysosomes.. J Cell Biol.

[36] Ganley IG, Carroll K, Bittova L, Pfeffer S (2004). Rab9 GTPase regulates late endosome size and requires effector interaction for its stability.. Mol Biol Cell.

[37] Doyotte A, Russell MR, Hopkins CR, Woodman PG (2005). Depletion of TSG101 forms a mammalian “Class E” compartment: a multicisternal early endosome with multiple sorting defects.. J Cell Sci.

[38] Johannsdottir HK, Mancini R, Kartenbeck J, Amato L, Helenius A (2008). Host cell factors and functions involved in Vesicular stomatitis virus entry.. J Virol.

[39] Abrami L, Lindsay M, Parton RG, Leppla SH, van der Goot FG (2004). Membrane insertion of anthrax protective antigen and cytoplasmic delivery of lethal factor occur at different stages of the endocytic pathway.. J Cell Biol.

[40] Wei X, Decker JM, Liu H, Zhang Z, Arani RB (2002). Emergence of resistant human immunodeficiency virus type 1 in patients receiving fusion inhibitor (T-20) monotherapy.. Antimicrob Agents Chemother.

[41] Melikyan GB, Barnard RJ, Abrahamyan LG, Mothes W, Young JA (2005). Imaging individual retroviral fusion events: from hemifusion to pore formation and growth.. Proc Natl Acad Sci U S A.

[42] Tobiume M, Lineberger JE, Lundquist CA, Miller MD, Aiken C (2003). Nef does not affect the efficiency of human immunodeficiency virus type 1 fusion with target cells.. J Virol.

[43] Sherer NM, Lehmann MJ, Jimenez-Soto LF, Ingmundson A, Horner SM (2003). Visualization of retroviral replication in living cells reveals budding into multivesicular bodies.. Traffic.

[44] Andrawiss M, Takeuchi Y, Hewlett L, Collins M (2003). Murine leukemia virus particle assembly quantitated by fluorescence microscopy: role of Gag-Gag interactions and membrane association.. J Virol.

[45] Kimpton J, Emerman M (1992). Detection of replication-competent and pseudotyped human immunodeficiency virus with a sensitive cell line on the basis of activation of an integrated beta-galactosidase gene.. J Virol.

[46] Sage D, Neumann FR, Hediger F, Gasser SM, Unser M (2005). Automatic tracking of individual fluorescence particles: Application to the study of chromosome dynamics.. Ieee Trans Image Process.

[47] Ewers H, Smith AE, Sbalzarini IF, Lilie H, Koumoutsakos P (2005). Single-particle tracking of murine polyoma virus-like particles on live cells and artificial membranes.. Proc Natl Acad Sci U S A.

