# Supplementary figures and images for: Synchronized Retrovirus Fusion in Cells Expressing Alternative Receptor Isoforms Releases the Viral Core into Distinct Sub-cellular Compartments
Source: PLoS Pathog. 2012 May 10;8(5):e1002694. doi: 10.1371/journal.ppat.1002694 (PMC3349758; doi:10.1371/journal.ppat.1002694)

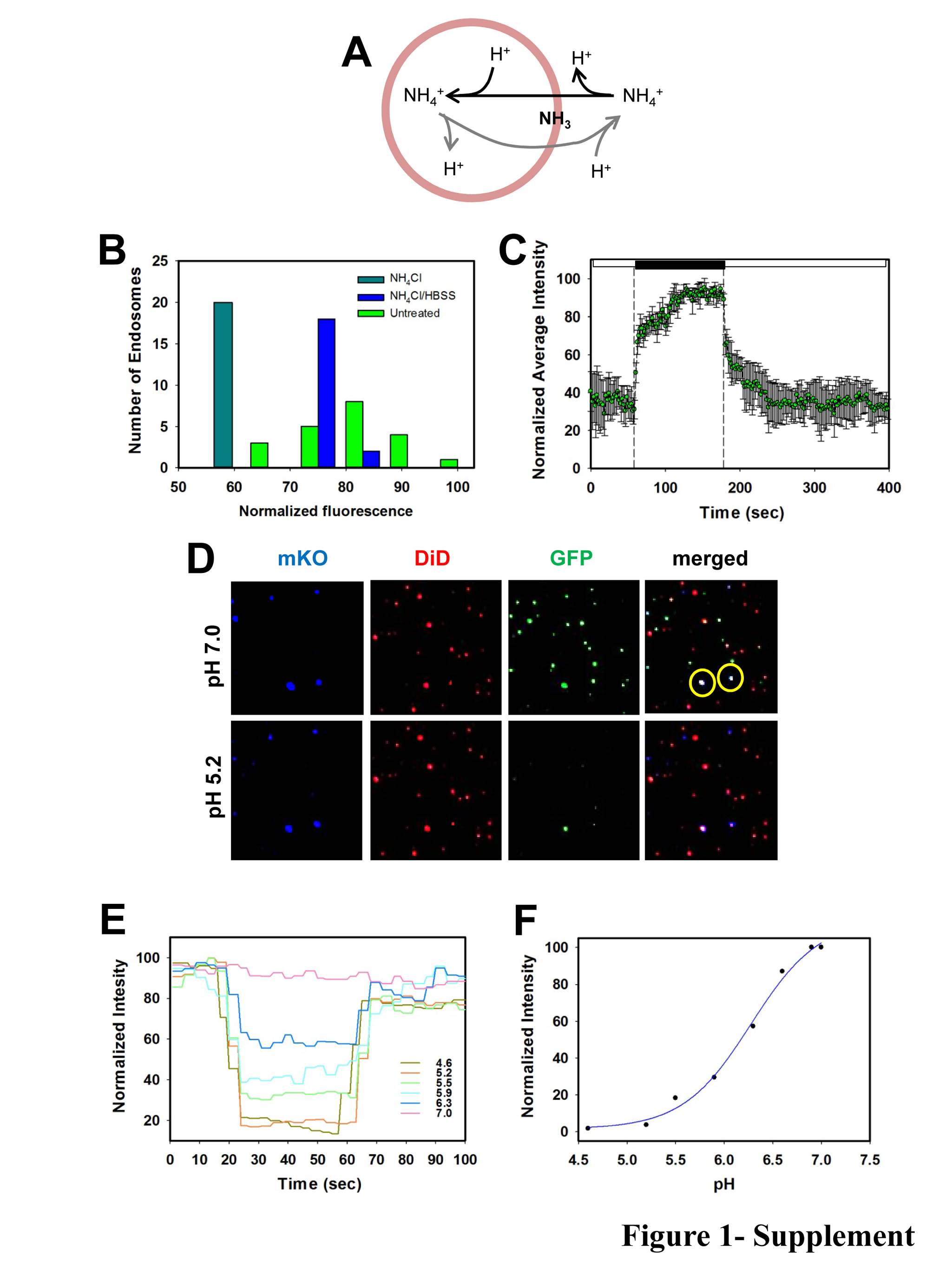

Supplement: Figure S1 — Measurements of endosomal pH using triple-labeled pseudoviruses. (A) Changes in pH upon addition/removal of NH4Cl. NH4 + permeates through a lipid membrane in its neutral ammonia form, which then acquires a proton and raises the pH of the target compartment. Conversely, NH4 + efflux leaves excess protons and thus lowers the pH. (B) Distributions of endosomal pH in CV-1 cells expressing TVA950. Cells were pre-loaded with 40 µg/ml of pHrodo dextran according to the manufacturer's instructions, and fluorescence emission at 585 nm was recorded. The fluorescence of pHrodo dextran increases at low pH [23], so acidic compartments are brighter than neutral endosomes. Total fluorescence intensity of single endosomes was measured and normalized to the maximum fluorescence intensity in control (untreated) cells. Graph shows the normalized distribution of fluorescence intensities of endosomes in untreated TVA950 cells (green bars), in the presence of 70 mM NH4Cl (following a 40 min-incubation at 37°C, dark cyan bar), and immediately after replacing NH4Cl with HBSS (NH4Cl/HBSS, blue bars). (C) Changes in the average fluorescence of five pHrodo dextran-laden endosomes in CV-1 cells upon perfusion with 70 mM NH4Cl in HBSS (white horizontal bar) with an intermittent perfusion with plain HBSS for 2 min (black horizontal bar). (D) Pseudoparticles carrying ASLV-A Env were co-labeled with MLV Gag-mKO (core), GFP-ICAM-1 (membrane) and DiD (membrane). Viruses were allowed to adhere to poly-L-lysine coated coverslips and imaged at room temperature in buffers adjusted to different pH values. Only particles positive for all three markers, Gag-mKO (blue), GFP-ICAM-1 (green) and DiD (red) where used for analysis (exemplified by two encircled white particles on the top right panel. The pH 5.2 citrate buffer strongly reduces the GFP-ICAM-1 fluorescence (lower panel) without affecting the reference DiD signal or Gag-mKO fluorescence. (E) Changes in the GFP fluorescence in response to citrate buf [file ppat.1002694.s001.tif]

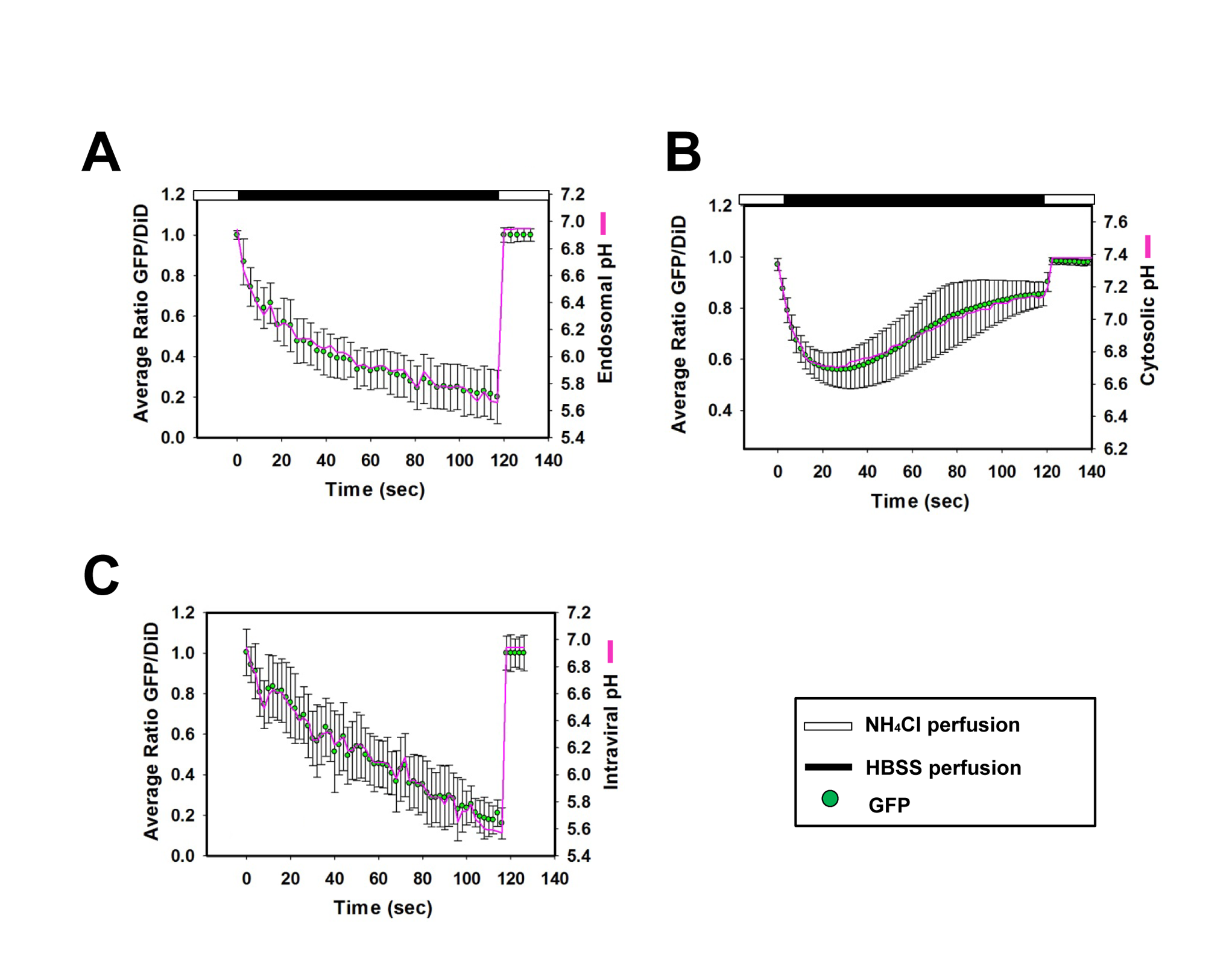

Supplement: Figure S2 — Dynamics of endosomal, cytosolic and intraviral pH upon removal of NH4Cl. (A) The average intensity profiles for non-fusogenic ASLV pseudoviruses labeled with GFP-ICAM-1, DiD and MLV Gag-mKO (pooled data using cells expressing TVA800 and TVA950). Cells were perfused with NH4Cl (white horizontal bar) followed by a 2 min-perfusion with HBSS (black bar). The changes in average fluorescence ratio of GFP over DiD of 20 randomly chosen particles following the NH4Cl removal/addition are shown (green circles). The average pH trace (pink line) was obtained from the calibration curve shown in Figure S1F. Error bars correspond to the standard deviation from the 20 GFP/DiD ratios was obtained for each time point. (B) TVA800 and TVA950 cells transiently expressing cytosolic eGFP were stained with DiD and imaged. The average ratio of GFP/DiD (green dots) was obtained for selected regions of interest within different cells. The corresponding error was obtained by calculating the standard deviation of each time point for every GFP/DiD measurement. The corresponding average pH trace (pink line) was obtained from the calibration curve shown in Figure S1F. The resting cytosolic pH value was set to 7.3, as shown in [25]. (C) Average decay in the intraviral Gag-GFP (plotted as Gag-GFP/DiD ratio) caused by removal of NH4Cl. Each datum point is obtained by averaging fluorescence ratios from 20 randomly chosen viral particles. The corresponding pH trace (pink line) was obtained from the calibration curve shown in Figure 1F. (TIF) [file ppat.1002694.s002.tif]

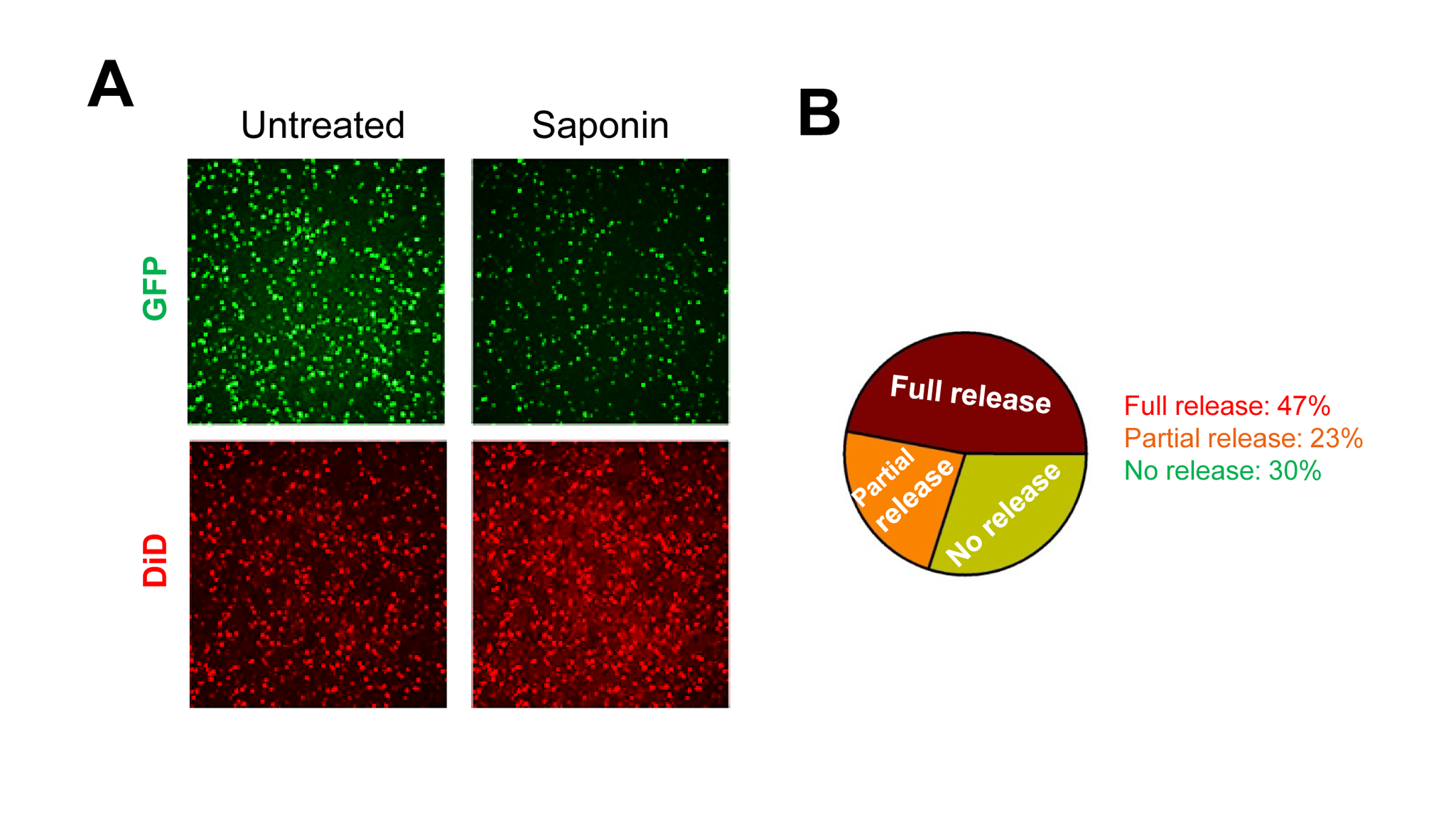

Supplement: Figure S3 — Analysis of releasable Gag-GFP pool in ASLV pseudoviruses. (A) ASLV pseudoviruses co-labeled with Gag-GFP/DiD were immobilized on coverslips and treated with 0.1 mM saponin at room temperature. Images were taken before and 2–3 min after exposure to saponin, which mediated a quick loss of green fluorescence puncta without noticeably affecting the DiD fluorescence. (B) Analysis for the particles before and after saponin treatment showed that 30% were completely resistant to lysis, 23% lost only a portion of their fluorescence, and 47% completely lost the GFP marker. The partial release phenotype was unlikely due to virus aggregation, since larger particles were excluded from analysis. (TIF) [file ppat.1002694.s003.tif]

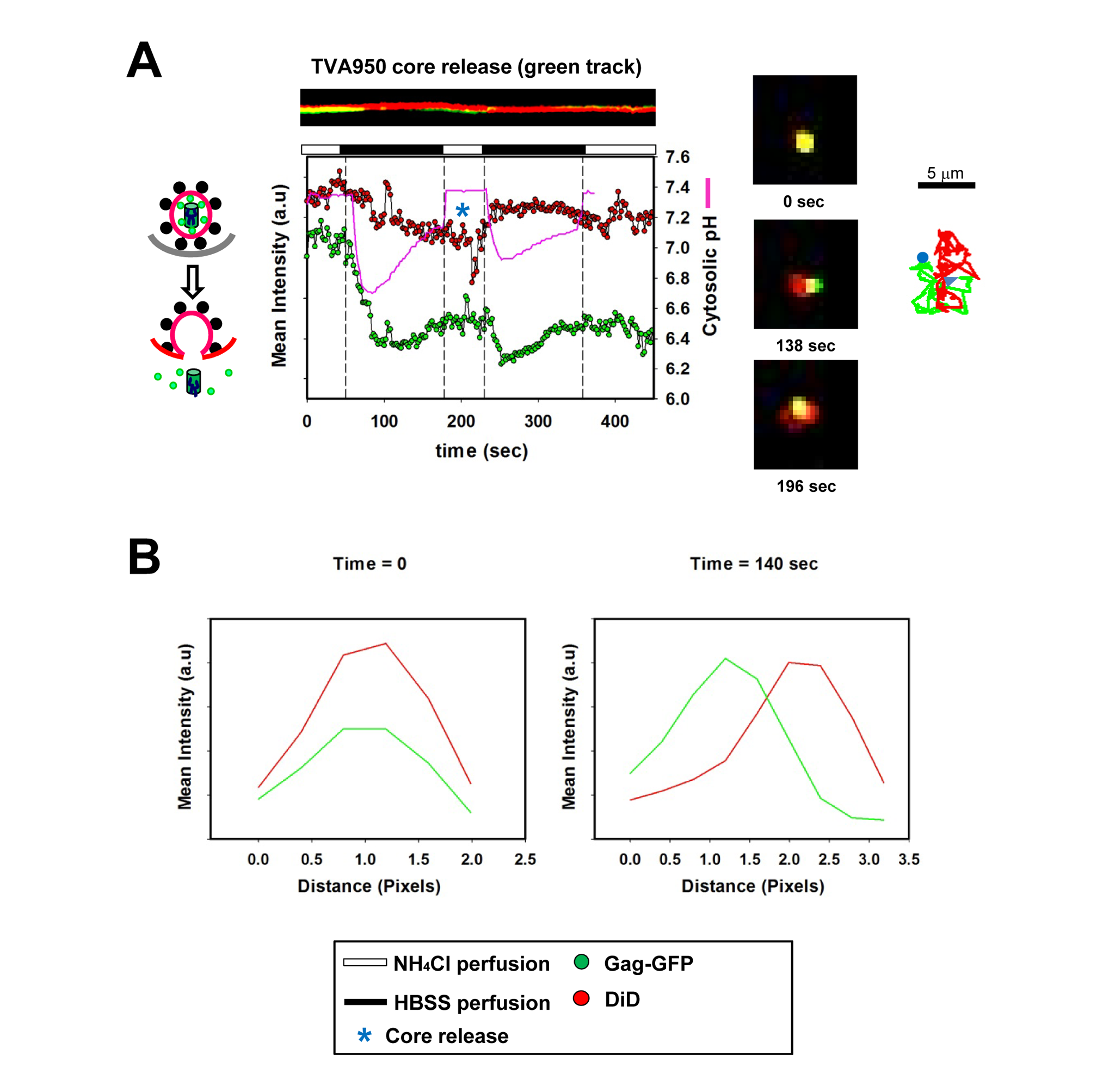

Supplement: Figure S4 — Incomplete separation of GFP- and DiD-labeled puncta during the NH4Cl arrest/release protocol. ASLV pseudoviruses co-labeled with Gag-GFP (green) and DiD (red) were subjected to the NH4Cl arrest/release protocol in TVA950 cells (see Materials and Methods for details). (A) In spite of the lack of full separation of green and red puncta (image panels), the GFP fluorescence changes (green dots) during both HBSS perfusion intervals paralleled the changes in the cytosolic pH (pink lines) under the same perfusion conditions. The right panel shows the trajectories of the SVP (green) and an endosome (red). The initial, fully overlapping part of the trajectory is colored blue. (B) Line histograms showing the spatial overlap of GFP-Gag (green line) and DiD (red) puncta before (t = 0, left) and after (t = 140 sec, right) partial SVP separation from an endosome. (TIF) [file ppat.1002694.s004.tif]

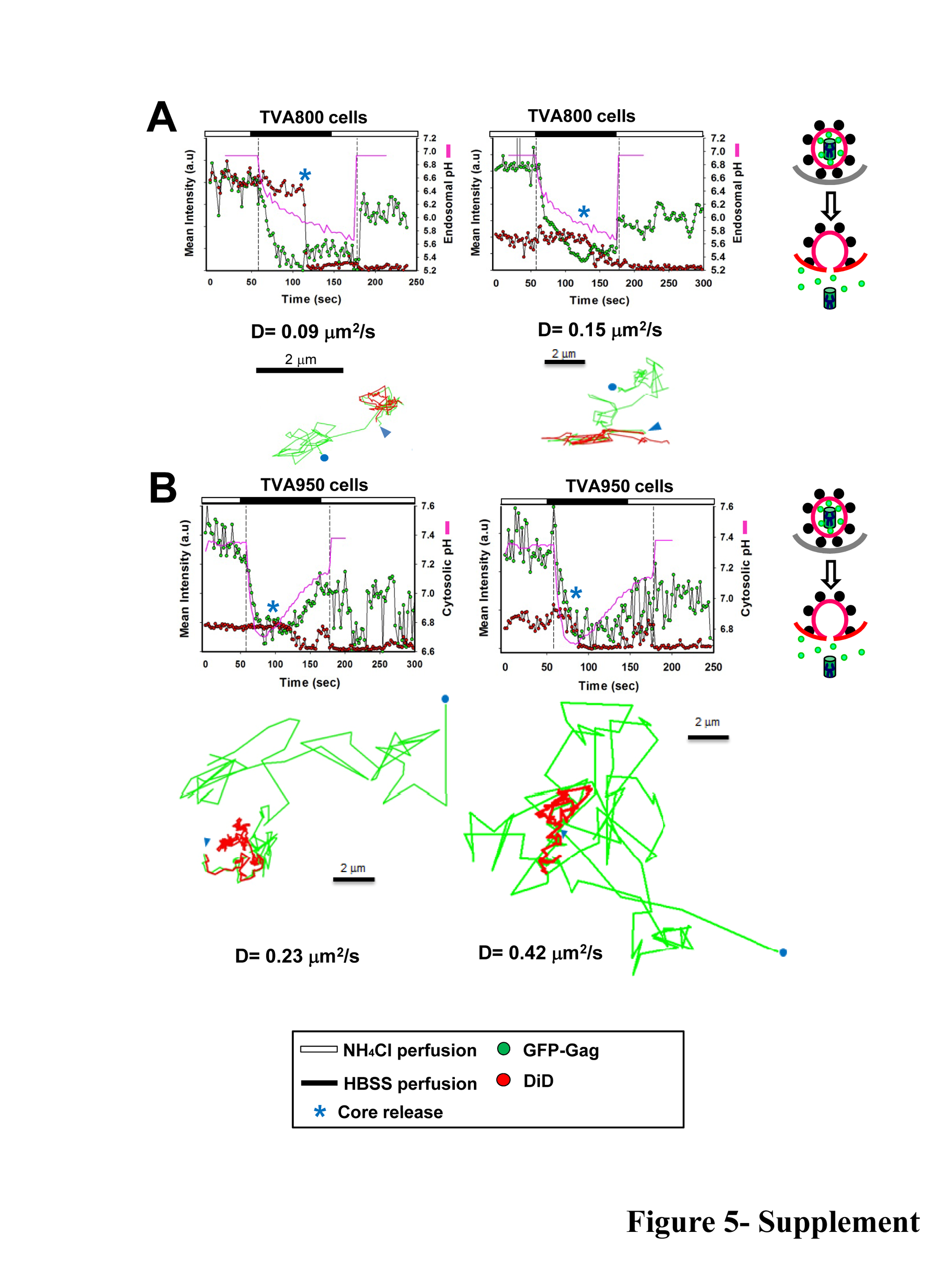

Supplement: Figure S5 — Examples of SVP release (single HBSS pulse). Two examples of spatial separation of GFP- and DiD-tagged puncta following the NH4Cl arrest/release protocol for TVA800 cells (A) and TVA950 cells (B). In all cases, SVP release occurred during HBSS perfusion (blue asterisk) and is detected by the abrupt drop in the DiD intensity signal (red circles). The cytosolic pH profile (pink lines in all panels) is also shown. In the case of TVA800 cells (A), the GFP fluorescence recovery occurs only after returning to NH4Cl (t = 180 sec), a behavior expected for endosomal compartments. In TVA950 cells the change in GFP-Gag intensity after SVP release paralleled that of cytosolic pH, suggesting that the viral capsid was released into the cytoplasm. The trajectories for the GFP-tagged SPVs (solid green line) together with the DiD-recipient vesicle trajectories (solid red line) are shown below each intensity profile. In all cases, the motilities of SVPs after their separation from endosomes were different from those of endosomes that received DiD. Clearly, the SPV movement is more restricted in TVA800 cells (A) than in TVA950 cells (B). Diffusion coefficients (calculated as described in Materials and Methods) shown next to the trajectories are 2-fold greater for SPVs tracked in TVA950 cells compared to TVA800 cells. (TIF) [file ppat.1002694.s005.tif]

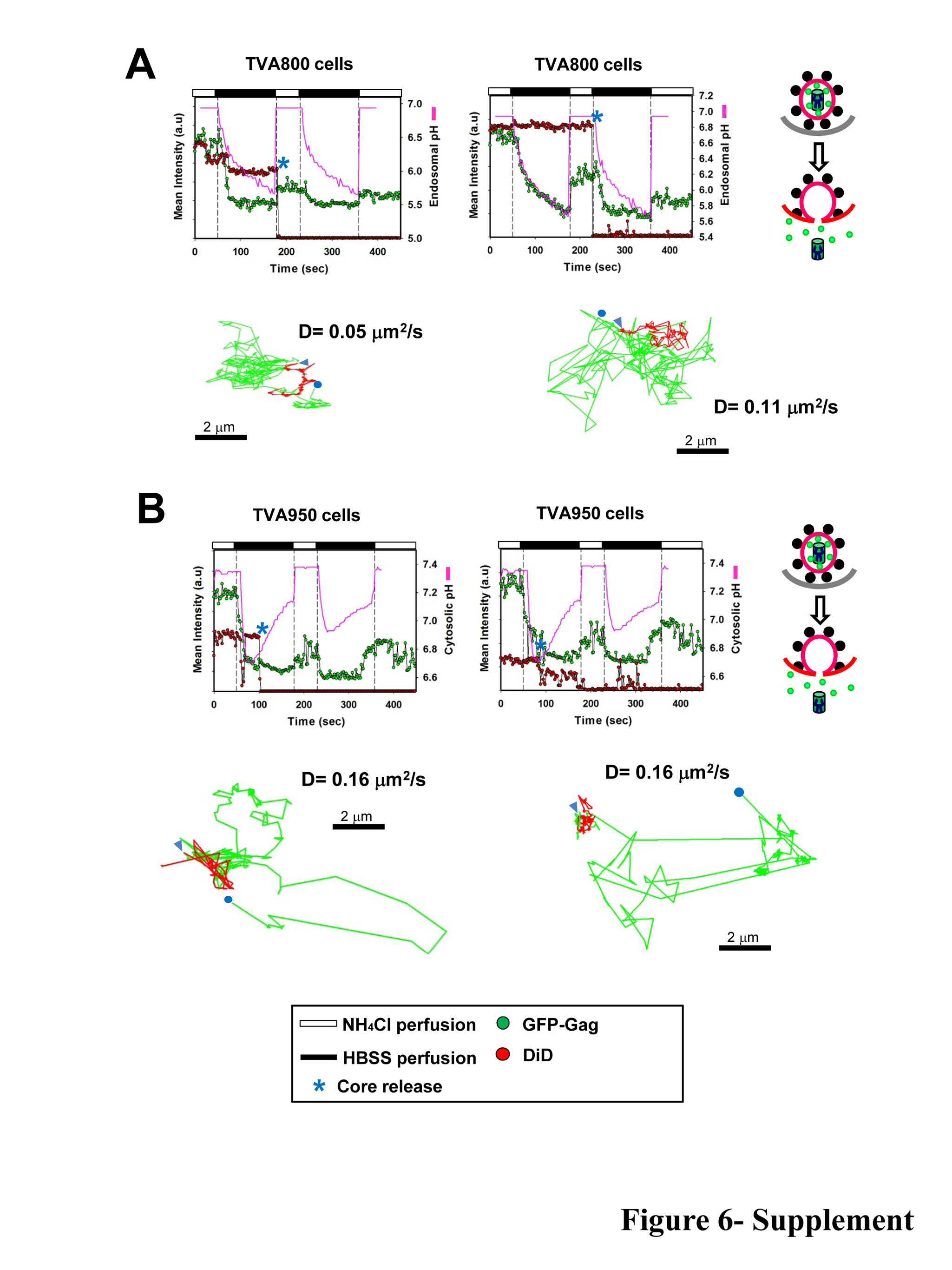

Supplement: Figure S6 — Examples of spatial SVP release (double HBSS pulse). Examples of delayed spatial separation of GFP- and DiD-tagged puncta following the NH4Cl arrest/release protocol in TVA800 (A) and TVA950 cells (B). SVPs were released after the first HBSS perfusion pulse, which ended at t = 180 sec. Therefore a second HBSS pulse was applied (between 240 and 360 sec) to assess the localization of the released cores. This protocol revealed different pH environment for the released capsids in cells expressing alternative TVA receptors. The pH profile of the cytosol (solid pink line in all panels) shows a fast recovery following the first and the second HBSS pulses. This behavior is matched by the GFP intensity profiles from SPVs (green dots) released into TVA950 cells (B), but not into TVA800 cells (A). The trajectories for the GFP-Gag (solid green line) signal together with the DiD (solid red line) are also shown under each intensity profile. Comparison of the SVP trajectories in TVA800 and TVA950 cells shows that their mobility was more restricted in TVA800 cells (see also Figure 3 and Figure S5). The diffusion coefficients calculated as described in the Materials and Methods are also given for the representative particles. (TIF) [file ppat.1002694.s006.tif]

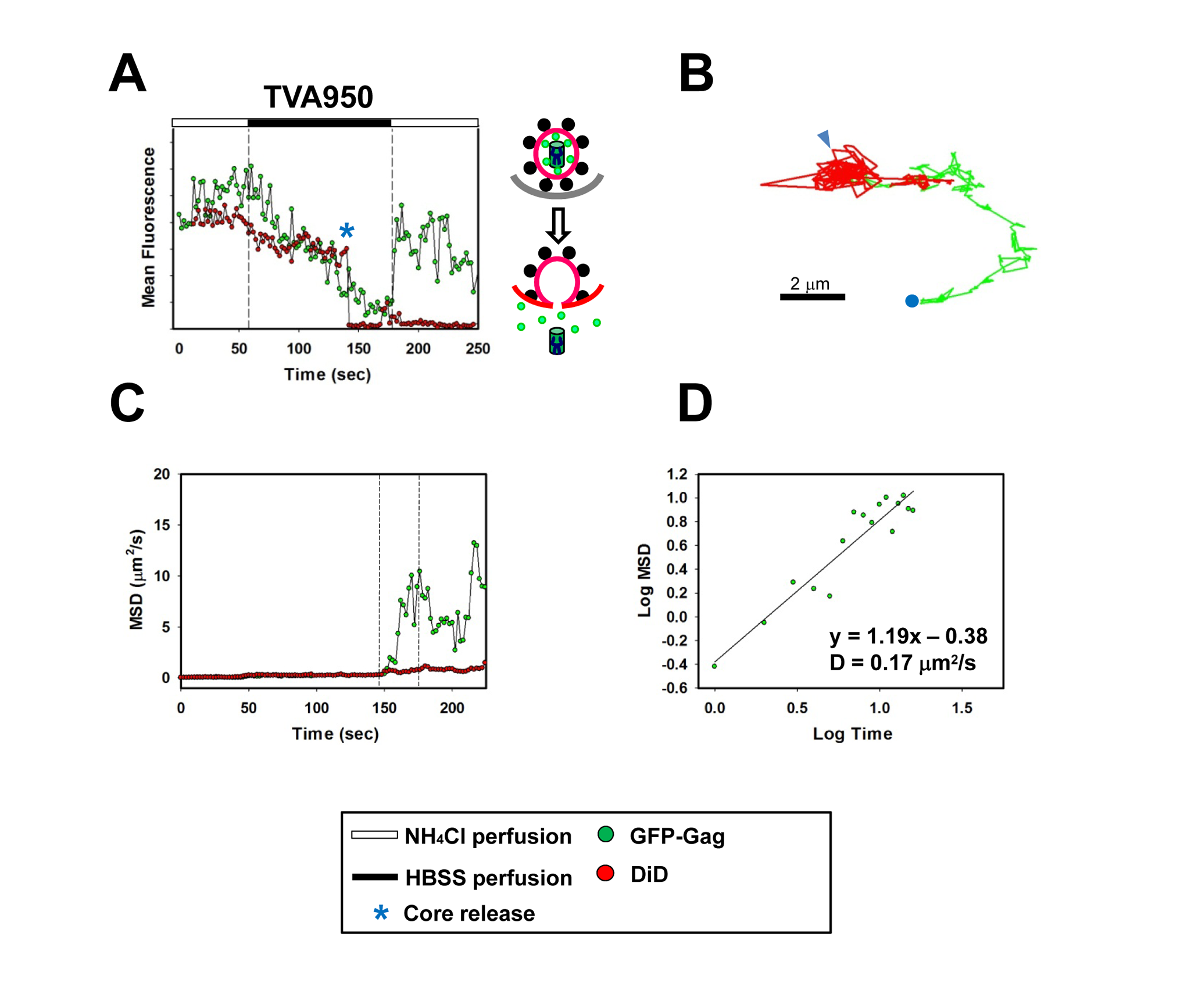

Supplement: Figure S7 — Example of core release into an endosome of a TVA950 cell. (A) Spatial separation of GFP- and DiD-tagged puncta in a TVA950 cell occurred during HBSS perfusion (blue asterisk) as evidenced by the abrupt drop in the DiD intensity (red circles). The GFP fluorescence recovers only after returning to NH4Cl (t = 180 sec), suggesting the SVP release into an endosomal compartment. The endosome and SVP trajectories are shown in B. The mean square displacement of SVP and DiD are also depicted in C. The linear part of the MSD after SVP release (between dashed lines in C) was chosen to generate a log-log plot for the MSD over time (D). The diffusion coefficient (D = 0.16 µm2/sec) of SVP was calculated using the y0 value determined from the intercept of a linear fit with the Y axis and applying Eq. 1 (see the main text for details). (TIF) [file ppat.1002694.s007.tif]

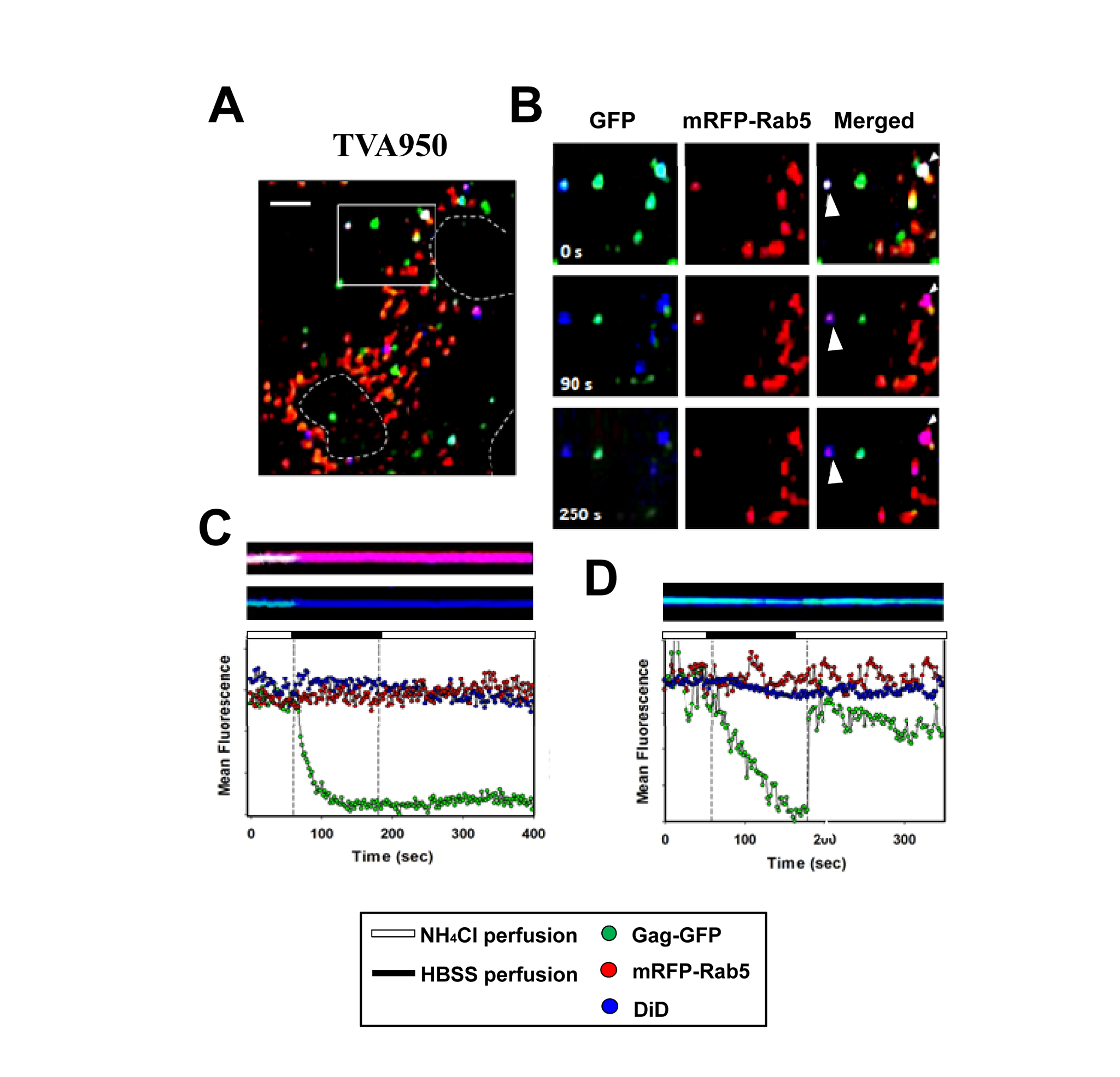

Supplement: Figure S8 — Single ASLV fusion with Rab5-positive endosome. TVA950 cells were transiently transfected with a marker for early endosomes, mRFP-Rab5 (A). ALSV pseudoviruses co-labeled with Gag-GFP (green) and DiD (blue) were internalized by cells in the presence of NH4Cl for 40 min at 37°C. Cells were initially perfused with 70 mM NH4Cl in HBSS followed by perfusion with plain HBSS for 2 min (black horizontal bars) and, finally, with NH4Cl. (A, B) Fusion with a Rab5-positive endosome in TVA950 cell is illustrated. Double-labeled viruses co-localized with mRFP-Rab5 endosomes (red) appear white. The white square in (A), delineates a region of interest magnified in (B) and shown for different time points corresponding to perfusion with different buffers t = 0 sec (NH4Cl), t = 90 sec (HBSS) and t = 250 sec (NH4Cl). The arrowheads in the merged panels mark two particles undergoing fusion in Rab5-positive endosomes. (C) Fusion was associated with a complete loss of GFP fluorescence (green circles) from virions during the HBSS perfusion, while the fluorescence intensities of DiD (blue circles) and mRFP-Rab5 (red circles) remained steady. The corresponding two-color kymograph (DiD/Gag-GFP) and three-color kymograph (DiD/Gag-GFP and mRFP-Rab5) are also shown. For comparison, the intensity profile for a non-fusogenic particle within a Rab5-positive endosome is shown in panel D along with the corresponding two-color kymograph (DiD/Gag-GFP). (TIF) [file ppat.1002694.s008.tif]
